# Database of Wannier tight-binding Hamiltonians using high-throughput density functional theory

**DOI:** 10.1038/s41597-021-00885-z

**Published:** 2021-04-13

**Authors:** Kevin F. Garrity, Kamal Choudhary

**Affiliations:** 1 Materials Science and Engineering Division, National Institute of Standards and Technology, Gaithersburg, Maryland 20899 USA; 2 Theiss Research, La Jolla, CA 92037 USA

**Keywords:** Topological matter, Electronic structure

## Abstract

Wannier tight-binding Hamiltonians (WTBH) provide a computationally efficient way to predict electronic properties of materials. In this work, we develop a computational workflow for high-throughput Wannierization of density functional theory (DFT) based electronic band structure calculations. We apply this workflow to 1771 materials (1406 3D and 365 2D), and we create a database with the resulting WTBHs. We evaluate the accuracy of the WTBHs by comparing the Wannier band structures to directly calculated spin-orbit coupling DFT band structures. Our testing includes k-points outside the grid used in the Wannierization, providing an out-of-sample test of accuracy. We illustrate the use of WTBHs with a few example applications. We also develop a web-app that can be used to predict electronic properties on-the-fly using WTBH from our database. The tools to generate the Hamiltonian and the database of the WTB parameters are made publicly available through the websites https://github.com/usnistgov/jarvis and https://jarvis.nist.gov/jarviswtb.

## Abstract (summary)

**Table:** 

Measurement(s)	material property • electronic band structure
Technology Type(s)	computational modeling technique
Factor Type(s)	material

Machine-accessible metadata file describing the reported data: 10.6084/m9.figshare.14161406

## Background & Summary

Wannier functions (WF) were first introduced^1^ in 1937, and have proven to be a powerful tool in the investigation of solid-state phenomenon such as polarization, topology, and magnetization^2^. Mathematically, WFs are a complete orthonormalized basis set that act as a bridge between a delocalized plane wave representation commonly used in electronic structure calculations and a localized atomic orbital basis that more naturally describes chemical bonds^2–8^. One of the most common ways of obtaining Wannier tight-binding Hamiltonians (WTBH)^9–11^ is by using the Wannier90 software package^12^ to generate maximally localized Wannier functions, based on underlying density functional theory (DFT) calculations. However, obtaining high-quality Wannier functions requires several choices by code users, including which bands and energy ranges to Wannierize, as well as a choice of starting orbitals. Therefore, in order to unlock the many materials properties that can be calculated with WTBH for use in high-throughput computations, we provide tools to automate the Wannierization of DFT band structures, and we generate a database of verified WTBH for use in future applications.

The computational advantage of Wannier functions comes from their localization, which allows the WTBH to be determined once on a relatively coarse real-space grid, and then Fourier transformed to obtain the Hamiltonian and its derivatives at arbitrary k-points in the Brillouin zone, allowing many expressions to be evaluated efficiently^13^. Many computationally expensive quantities such as the Z_2_ index, Chern number, Fermi-surface, Weyl-chirality, Hall conductivity, spin-texture, photo-galvanic effect, thermoelectric coefficients, thermal properties, Landau level applications, gyrotropic effects, and shift-photocurrent^12,14–19^ can be efficiently computed with WTBHs. In addition, many materials properties are based on localized phenomena^20–22^ such as impurities^23^, defects^24,25^, excitons^26^, polarons^27^, screened electron-electron interaction^28^, and electron-phonon interactions^29^, all of which can be modeled in a Wannier basis^30^. In addition, an examination of the Wannier Hamiltonian can provide intuition to help understand bonding that is difficult to get from examining the delocalized Kohn-Sham eigenvectors directly. They are also useful in second quantization based beyond-DFT calculations such as Dynamical Mean Field Theory (DMFT)^31,32^.

Since its launch in 2011, the Materials Genome Initiative (MGI)^33^ has spurred the generation of several high-throughput databases and tools such as from AFLOW^34^, Materials Project^35^, Open Quantum Materials Database (OQMD)^36^, Materials Cloud^37^, AiiDA^38^, NOMAD^39^, and NIST-JARVIS^40^. They have played key roles in the generation of electronic-property related databases to reduce the time between materials discovery and application. However, the development of WTBH databases and tools are still in the developing phases^41–46^.

Since the work of Souza, Marzari, and Vanderbilt (SMV)^2,5–7^, which requires an initial guess of the Wannier subspace and a minimization procedure to achieve maximum localization, there have been several methods proposed for determining localized Wannier functions with less human intervention. One method that has been applied in a high-throughput manner is the AFLOWπ projection method^41,42^, which uses a projection of the Bloch states on localized atomic orbitals without minimization to construct a localized basis. A second is the Selected Columns of the Density Matrix (SCDM) method, which constructs a localized subspace without an initial guess, based on properties of the density matrix^44,45^. In this work, we instead use the original method of SMV, but we develop a workflow that can automatically construct the initial guess and set various parameters needed for Wannierization, as well as test the resulting WTBH.

The goal of this paper is to: a) develop a high throughput workflow for Wannierization of DFT calculations, b) develop a database of verified Wannier-based tight-binding Hamiltonians along with all related input/output files, c) develop web-apps for convenient WTBH predictions. We use our Wannierization workflow on the JARVIS-DFT (https://jarvis.nist.gov/jarvisdft) database which is a part of the MGI at NIST. The NIST-JARVIS^40^ (https://jarvis.nist.gov) has several components such as JARVIS-FF^47,48^, JARVIS-DFT^48–57^, JARVIS-ML^49,51,57–59^, JARVIS-STM^51^, JARVIS-Heterostructure^53^ and hosts material-properties such as lattice parameters^50^, formation energies^60^, 2D exfoliation energies^55^, bandgaps, elastic constants^50^, dielectric constants^59^, infrared intensities^59^, piezoelectric constants^59^, thermoelectric properties^57^, optoelectronic properties, solar-cell efficiencies^47,49^, topological materials^17,21^, electric field gradient^61^, and computational STM images^51^. The JARVIS-DFT database consists of ≈ 40000 3D and ≈1000 2D materials. As an initial step, we deploy our computational workflow on the materials that were recently predicted to be topologically non-trivial based on the spin-orbit spillage technique, including three dimensional (3D), two dimensional (2D), magnetic, non-magnetic, insulating, and metallic systems^52,60^ including spin-orbit interactions. After obtaining the WTBH from DFT, we perform several checks to ensure the quality of the Hamiltonians. Although here we present results mainly for high-spillage materials, we will be extending this workflow to the entire JARVIS-DFT database. Currently, we have calculated Wannier Hamiltonians including spin-orbit coupling for 1406 3D and 365 2D materials, which can be used to efficiently calculate materials properties using either our software tools or other external software such as Wannier-tools^14^, Z2Pack^62^, WOPTIC^63^, EPW^64^. We believe that releasing this database and toolset for use by the materials community should enable accelerated materials prediction and analysis.

## Methods

**Fig. 1:**
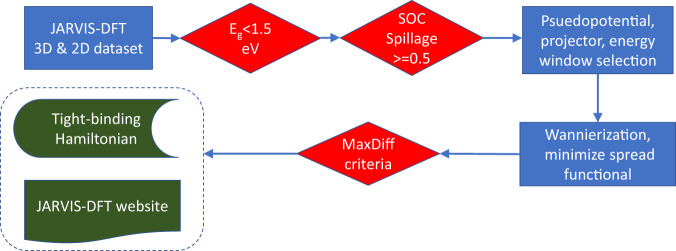
Workflow showing the Wannierization from using the DFT calculations.

The methodology supporting the current project consists of several steps that are given in Fig. 1. The beginning of the procedure selects materials for Wannierization that we have prescreened to have strong spin-orbit coupling effects in our previous work and are therefore likely to be topological insulators or semimetals^52,60^. The main subject of the current work is the automation of the Wannierization, which proceeds by first selecting parameters for the Wannierization, including the initial guess for the Wannier functions and a “frozen window.” We then perform the Wannierization and test the resulting WTBH. These steps are discussed in detail below.

DFT calculations were carried out using the Vienna Ab-initio simulation package (VASP)^65^ software using the workflow given on our JARVIS-Tools github page (https://github.com/usnistgov/jarvis)^66^. We use the OptB88vdW functional^67^, which gives accurate lattice parameters for both vdW and non-vdW (3D-bulk) solids^50,55^. We optimize the crystal-structures of the bulk and monolayer phases using VASP with OptB88vdW. Because spin-orbit coupling (SOC) is not currently implemented for OptB88vdW in VASP, we carry out spin-orbit PBE calculations. Such an approach has been validated by Refs. ^60,68^. The crystal structure was optimized until the forces on the ions were less than 0.01 eV/Å and energy less than 10^−6^ eV. We use Wannier90^12^ to construct Maximally-Localized Wannier Functions (MLWF) based TB-Hamiltonians.

The basic formalism of Wannierization is well-established. We briefly review some aspects here, interested readers can see longer discussions in^5,19^. For a set of Bloch eigenvectors $\left|{\psi }_{n,k}\right\rangle$, a general set of WFs $\left|{\bf{R}}n\right\rangle$ (n = 1…N) can be written as:

1 $$\left|{\bf{R}}n\right\rangle =\frac{V}{{\left(2\pi \right)}^{3}}{\int }_{BZ}^{BZ}{\sum }_{m=1}^{N}{U}_{mn}^{({\bf{k}})}\left|{\psi }_{mk}\right\rangle {e}^{-i{\bf{k}}.{\bf{R}}}d{\bf{k}}$$

where **R** labels the unit cell of the WF, *V* is the volume of the unit cell, and ${U}_{mn}^{({\bf{k}})}$ is an arbitrary unitary matrix. To construct maximally-localized WFs, ${U}_{mn}^{({\bf{k}})}$ is chosen to minimize the following spread functional:

2 $$\varOmega ={\sum }_{n}[{\langle {r}^{2}\rangle }_{n}-{\bar{r}}_{n}^{2}]$$

where ${\bar{{\bf{r}}}}_{n}=\left\langle 0n\right|{\bf{r}}\left|0n\right\rangle$ and ${\langle {r}^{2}\rangle }_{n}=\langle 0n| {r}^{2}| 0n\rangle$. The minimization proceeds iteratively, based on an initial guess of localized orbitals.

For the case of interest in this work, we wish to describe both the valence and conduction bands near the Fermi level. Therefore, it is necessary to first select a set of bands to Wannierize, and to separate these bands from the free-electron-like bands that overlap energetically with the conduction bands^62^. The procedure to determine this localized subspace of Bloch wavefunctions proceeds similarly to minimization described above, where after an initial guess, the subspace is iteratively updated in order to minimize the spread function in Eq. 2. After this initial disentanglement step, the Wannierization of the selected subspace proceeds as described above.

Due to the iterative non-linear minimization employed during both the disentanglement and Wannierization steps, the localization and utility of the final Wannier functions depend in practice on the initial choice of orbitals that are used to begin the disentanglement procedure, and which are then used as the initial guess for the Wannierization. Our initial guesses consist of a set of atomic orbitals we have chosen to describe all the chemically relevant orbitals for each element in typical elemental systems and compounds. We provide the list of the orbitals we select for each element in Table S1. For many specific materials, it may be possible to select a smaller set of orbitals while still maintaining high-quality WFs that describe the bands of interest; however, our fairly inclusive set of orbitals is able Wannierize nearly all compounds in a high-throughput manner without human intervention. Because most applications of WFs are computationally inexpensive compared to the DFT calculations used to construct the WFs, in practice, our larger Wannier basis has only minimal computational cost. However, it is necessary to have enough empty bands in the underlying DFT calculation such that any empty orbitals chosen are included in the Bloch basis. We do not include any semicore orbitals in our Wannier basis, as they are generally well-separated in energy from the valence orbitals and are not necessary to describe bands near the Fermi level.

During the disentanglement step, it is possible to choose an energy range that is included exactly (“the frozen window”)^12^, with the result that the Wannier band structure will exactly match the DFT band structure in this energy range and at the grid of k-points used in the Wannierization (see discussion in Sec. 2.I in Ref. ^2^). We use a default frozen window of ±2 eV around the Fermi-energy. This window ensures that bands near the Fermi level are well described by the WTBH. Outside the frozen window, disagreement will tend to increase, as the procedure will select the most localized set of Wannier functions possible given the frozen window constraint, rather than reproduce additional bands exactly. This disagreement outside the frozen window should not affect most properties computed using Wannier interpolation, which depend on bands near the Fermi level, but other choices may work better for some applications. For cases where the original WFs were unsatisfactory (see below), we found that lowering the lower bound of this window to include all the valence bands often improves that WTBH, and we use this as a second possible Wannierization setting.

In order to validate our WTBH, we calculate the maximum absolute difference (*μ*) between the Wannier and DFT eigenvalues within an energy range of ±2 eV around the Fermi level:

3 $$\mu ={\,}_{\,\,n{\bf{k}}}^{max}\left(\left|{E}_{n{\bf{k}}}^{DFT}-{E}_{n{\bf{k}}}^{WTB}\right|\right)$$

As discussed above, at the grid of k-points used in the construction of the WFs and within the frozen window, the eigenvalues should match exactly by construction. Therefore, we require a different set of k-points to meaningfully test the WTBH. We choose to evaluate Eq. 3 on the dense lines of k-points along high symmetry directions that we already use to generate band structures. A weakness of this evaluation method is that highly dispersive energy bands (high $\frac{d{E}_{nk}}{dk}$) can result in high *μ* values even if the WTBH is of good quality because any slight shift in the *k*-direction of a dispersive band will result in a large energy error. We consider that systems with *μ* less than 0.1 eV to useful for most applications, and we provide data for the user to evaluate individual WTBH for their own applications.

Another failure mode for the Wannierization can be because the initial guess does not describe the DFT wavefunctions included in the Wannierization. This can happen either because important orbitals near the Fermi level were neglected, or if orbitals that were included have energies above the energy range included in the DFT calculation. However, as demonstrated below, we find that our chosen set of initial orbitals works well in most cases.

## Data Records

After the calculations, the TB Hamiltonians, Wannier90 input and outputs files are stored as tar files and distributed through the Figshare repository^69^. Each ‘zip’ file consists of wannier90.win, wannier90.wout, wannier90_hr.dat files. The wannier90.win and wannier90.wout are the input and output files for Wannier90 code respectively. The wannier90_hr.dat file can be loaded as WanHam class with scripts in the JARVIS-Tools (https://github.com/usnistgov/jarvis) and similar packages to apply post-processing analysis such as calculating band-structures. There are also a JavaScript Object Notation (JSON) and Portable Network Graphic (PNG) file for comparing DFT bandstructure to WTBH.

## Technical Validation

**Fig. 2:**
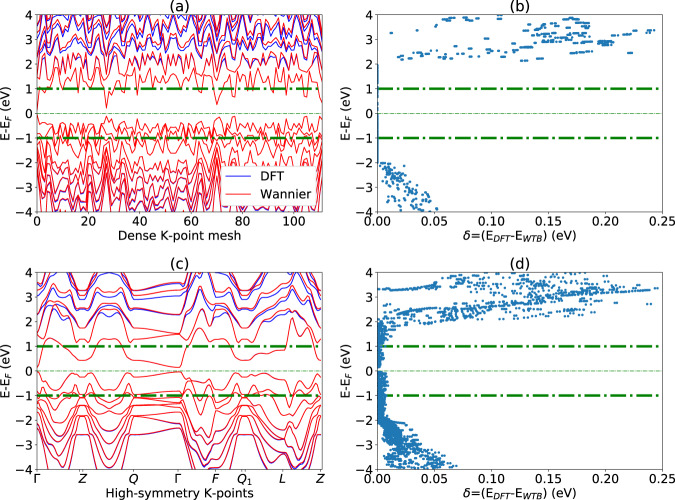
Comparison of DFT and WTB bandstructures for Bi_2_Se_3_. (**a**,**b**) on dense k-grid, (**c**,**d**) high-symmetry Brillouin zone points.

To validate the WTBHs generated in this work, we compare the Wannier electronic bands with directly calculated DFT bands and measure the differences using Eq. 3 on two different k-point grids. As an example, in Fig. 2, we show an evaluation of the WTBH for Bi_2_Se_3_. In this figure, the top two panels show the WTBH evaluated on the same k-point grid used to generate the WFs, while the lower two panels show the evaluation on a typical set of high-symmetry k-points and lines, which includes k-points not used in the construction of the WFs. Figure 2a,c show the eigenvalue comparison at separated k-points, with the WTBH bands in red and the DFT bands in blue, while Fig. 2b,d show the eigenvalue differences as a function of energy.

As expected, the agreement within the frozen window and on the dense k-point grid is almost exact, but quickly increases up to 0.25 eV when leaving the window. We find a larger but still small energy difference on the high symmetry grid Fig. 2c,d, with a maximal error in the frozen window of 9 meV. This test shows that this WTBH can be used to interpolate the band structure accurately.

**Fig. 3:**
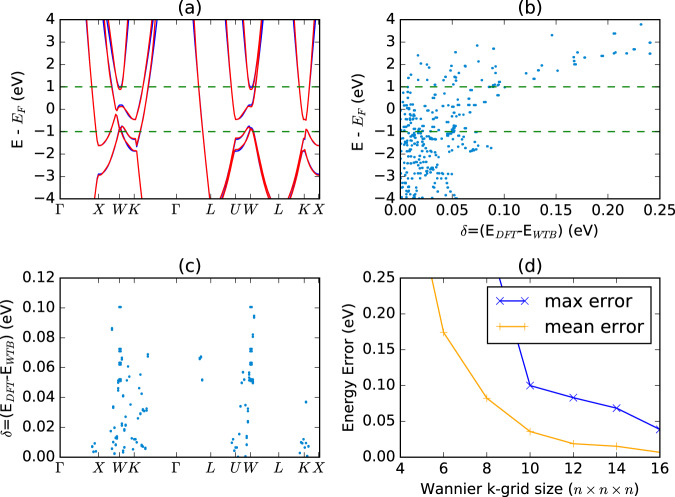
Comparison of DFT and WTBH bandstructures for Al (JVASP-816) on high-symmetry Brillouin zone. WTBH in (**a**–**c**) calculated using a 10 × 10 × 10 k-point grid. a) Band structure b) Energy error vs. DFT energy (eV) (**c**) Energy error (eV) at each k-point for eigenvalues with ± 1 eV of the Fermi level. (**d**) Maximum (blue) and mean (orange) energy error (eV) for WTBH made with different k-grids.

Next, we consider the Wannierization of *fcc*-Al, a free-electron-like metal that is more difficult to Wannierize. In Fig. 3a–c, we show a comparison between the DFT and WTBH bands constructed using a 10 × 10 × 10 k-point grid during the Wannierization, which is the value used in our workflow (Fig. 1). We find very good agreement for the general band shape. However, in Fig. 3b,c, we show a detailed look at the error near the Fermi level, finding maximum errors of nearly 0.1 eV, with large errors occurring where dispersive bands cross the Fermi level, such as between Γ and L. This higher error is due to the longer-range behavior of wavefunctions near the Fermi level in metals, as compared to the exponential decay in insulators^70,71^, causing the Wannierization to require a higher density of k-points to converge. In Fig. 3d, we show the maximum and average errors for WTBH constructed with different k-point grids. While the average error decays reasonably quickly, the maximum error requires a very dense mesh to converge. During a high-throughput study, it is necessary to make reasonable tradeoffs between convergence and computational time. Therefore, we pick reasonable convergence parameters for our Wannierization and report an error assessment for each WTBH, allowing users to assess the suitability of each WTBH for their applications.

**Fig. 4:**
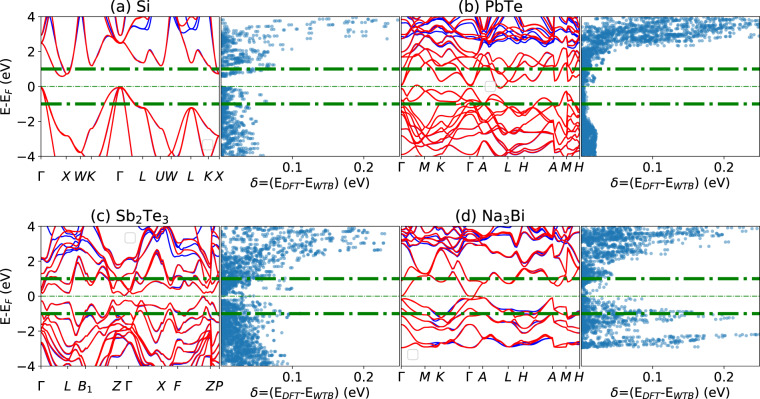
Examples of Wannier and DFT bandstructure and their energy difference plot for example 3D materials. (**a**) Si, (**b**) PbTe, (**c**) Sb_2_Te_3_, and (**d**) Na_3_Bi.

We show a few more examples of 3D WTBH in Fig. 4 for Si, PbTe, Sb_2_Te_3_ and Na_3_Bi, this time focusing only on the difference for the high-symmetry k-point grids. Similar to the Bi_2_Se_3_ case discussed above, they show the minimal difference, and the WTBH are able to reproduce features such as the Dirac point band crossing of Na_3_Bi between Γ and A.

**Fig. 5:**
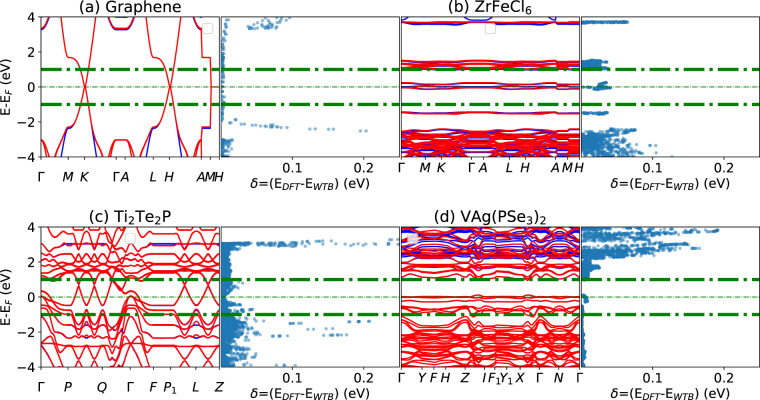
Examples of Wannier and DFT bandstructure and their energy difference plot for example 2D materials. (**a**) for graphene, (**b**) for ZrFeCl_6_, (**c**) for Ti_2_Te_2_P, and (**d**) for VAg(PSe_3_)_2_.

Bi_2_Se_3_, shown in Fig. 2, is a classic example of a 3D topological insulator. We show similar examples of 2D topological materials for graphene, ZrFeCl_6_, Ti_2_Te_2_P, and VAg(PSe_3_)_2_ in Fig. 5. A detailed topological analysis of these materials can be found in our previous works^60^. Similar to the Bi_2_Se_3_ case, we observe that the DFT and WTBH bands overlap within the ±2 eV window and start to separate for outside these ranges. We again find excellent agreement between the DFT and the Wannier bands. Similar figures will be available for all the WTBH produced in this work on our website, so that the user can evaluate the WTBH for their own applications.

**Fig. 6:**
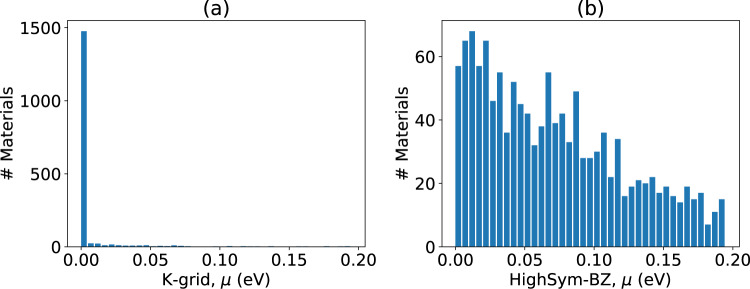
DFT-TB maximum difference (μ) distribution for all the Wannier Tight-binding Hamiltonians (WTBHs). (**a**) on a regular k-point grid, (**b**) on high-symmetry k-points.

As is clear from the above examples, it is important to evaluate the energy difference between the DFT and WTBH bands to ensure a high-quality Wannierization. We use the maximum value of these differences (MaxDiff) for each k-point and in the disentanglement window range (±2 eV) as the measure of the quality of WTBHs (see Eq. 3). We calculate these differences for both the k-point grid and high-symmetry BZ points. Choosing a tolerance of 0.1 eV as the maximum energy difference, we find that 93.0% of materials have a dense k-mesh MaxDiff less than the tolerance, while only 64% of materials have high-symmetry BZ MaxDiff less than the tolerance as shown in Fig. 6a,b respectively. These larger discrepancies mainly occur for metallic systems such as Al, which have very dispersive electronic bands that naturally result in larger errors as discussed earlier (see Fig. 3). In the supplementary section (Table S2), we include the MaxDiffs of all materials we tried to Wannierize to help demonstrate the utility and limitations of this high-throughput approach.

**Fig. 7:**
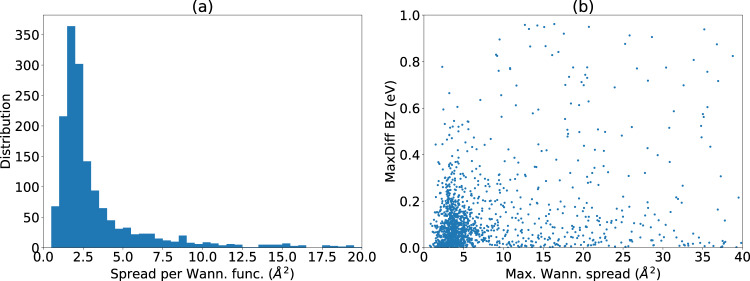
Analysis of Wannier function spread. (**a**) distribution of average Wannier function spread for every material, (**b**) comparison of maximum Wannier spread and the maximum Wannier and DFT band energy difference in a material.

In Fig. 7 we analyze the Wannier spread following Eq. 2 for all the materials in the database. We find that most of the Wannier orbitals are well-localized with average spread of less than 3 Å^2^. We do find a long tail of high Wannier spread in Fig. 7a. However, in Fig. 7b, we find little relationship between the Wannier spread and the accuracy of Wannier tight binding bands versus DFT. While high spread orbitals can indicate a failure of Wannierization, they can also be a result of including high energy states in the Wannierization, and these high spread Wannier functions may not affect the bands near the Fermi level.

**Fig. 8:**
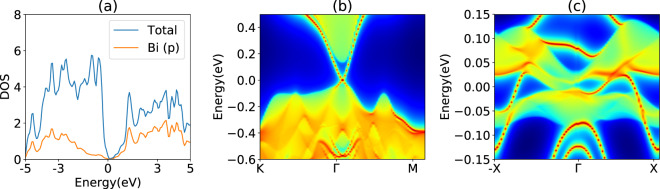
A few example applications of the WTB Hamiltonians. (**a**) total and projected density of states, (**b**) (001) surface band-structure of Bi_2_Se_3_, (**c**) edge bandstructure of VAg(PSe_3_)_2_.

Next, we show a few example applications to demonstrate the usefulness of the WTB Hamiltonians. In Fig. 8a, we show the total and Bi (p) projected density of states in the Bi_2_Se_3_ system. The DOS can be evaluated with a very dense k-point grid at low computational cost using WFs, allowing detailed features to be converged. As mentioned in the introduction section, the WTB Hamiltonians can also be used to study defect phenomenon, especially if the defect only removes weak vdW bonds. For example, in Fig. 8b, we show the (001) surface bandstructure of Bi_2_Se_3_. As expected for a Z_2_ topological insulator, there is a bulk gap and a surface Dirac cone feature at Γ. Similarly, we show the edge band structure of a 2D monolayer of VAg(PSe_3_)_2_ with ferromagnetic spin ordering. VAg(PSe_3_)_2_ is a 2D Chern insulator^60^, and the resulting spin-polarized conducing edge channel can be visualized in Fig. 8c.

**Fig. 9:**
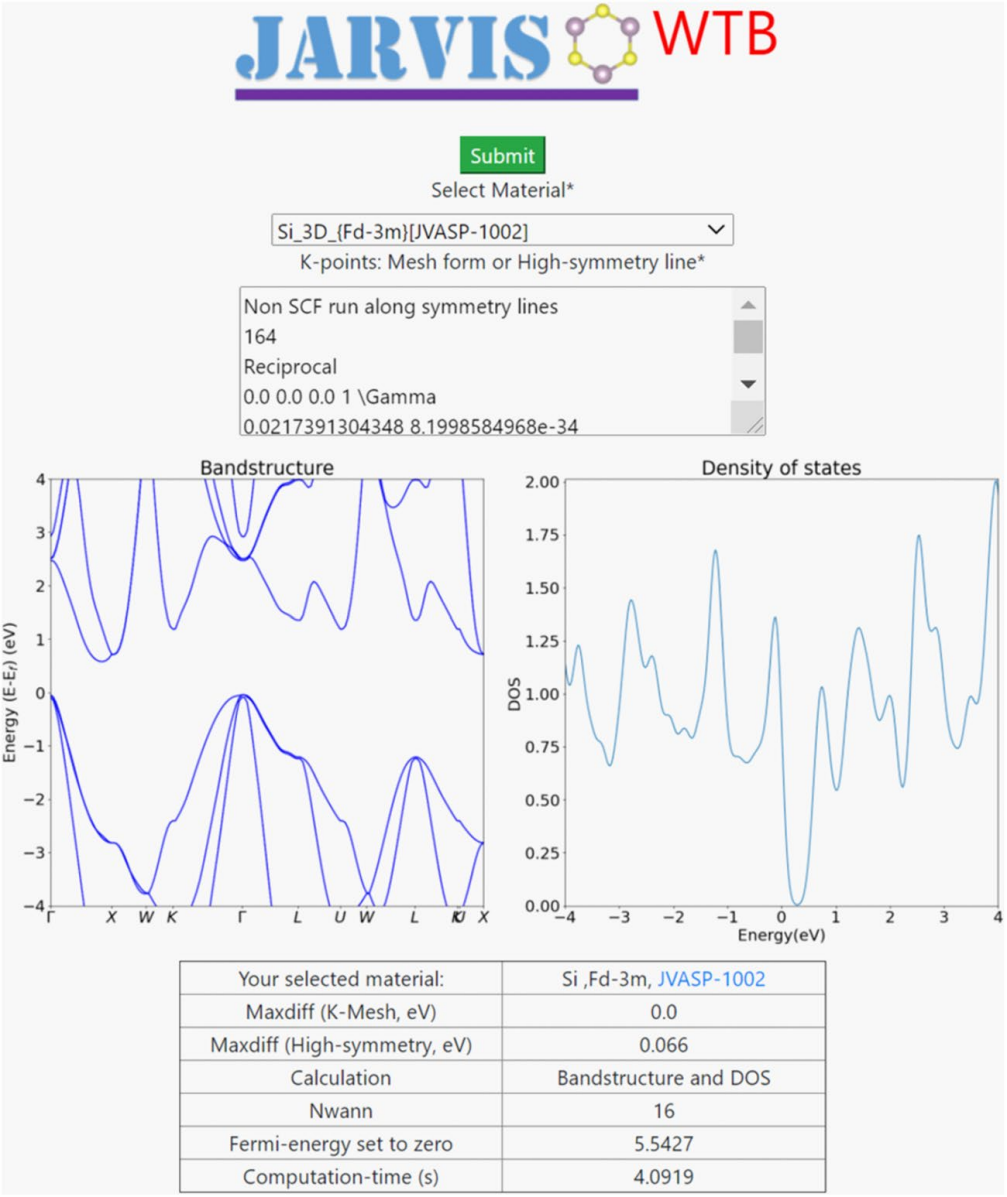
Snapshot of the web-app available at https://jarvis.nist.gov/jarviswtb/.

Finally, in Fig. 9 we show a screenshot of a web-app we are developing to allow users to calculate materials properties using WTBH directly from our database, without downloading the Hamiltonians themselves. We curate the list of materials on the app to only include materials with MaxDiff <0.1 eV, but all of the WTBH are available to download. Currently, we support the calculation of Wannier-projected band structures for arbitrary k-points, as well as projected DOS. In addition, we provide plots to evaluate the accuracy of the WTBH. We plan to add other WTBH related functionalities in the app soon.

## Usage Notes

The database presented here represents the largest collection of consistently calculated Wannier tight binding Hamiltonians of materials using density functional theory assembled to date. We anticipate that this dataset, and the methods provided for access will provide a useful tool in fundamental and application-related studies of materials. Our actual DFT verification provides insight into understanding the applicability and limitation of our the WTBH data. The WTBH can be used to obtain important electronic properties such as band-structures, density of states, and topological invariants in a computationally efficient way. Data-analytics tools can also be applied on the generated dataset.

## Supplementary information

Supplementary information

## Data Availability

Python-language based scripts for obtaining and analyzing the dataset are available at https://github.com/usnistgov/jarvis.
