# Supplementary material for: Database of Wannier tight-binding Hamiltonians using high-throughput density functional theory
Source: Sci Data. 2021 Apr 13;8:106. doi: 10.1038/s41597-021-00885-z (PMC8044170; doi:10.1038/s41597-021-00885-z)
Supplement: Supplementary file 1 — Supplementary information [file 41597_2021_885_MOESM1_ESM.docx]

**Supplementary information: Database of Wannier tight-binding Hamiltonians using high-throughput density functional theory**

Kevin F. Garrity^1^, Kamal Choudhary^1,2^

1 Materials Science and Engineering Division, National Institute of Standards and Technology, Gaithersburg, Maryland 20899, USA.

2 Theiss Research, La Jolla, CA, 92037, U.S.A.

Table S1: Semi-core states used during Wannierization. Note that several lanthanides and actinides are avoided.

| Element | POTCARs | N_electrons | Excluded_electrons | Projections | N_Wan_projections |
| --- | --- | --- | --- | --- | --- |
| Ag | Ag | 11 | 0 | s_d | 6 |
| Al | Al | 3 | 0 | s_p | 4 |
| Ar | Ar | 8 | 0 | s_p | 4 |
| As | As | 5 | 0 | s_p | 4 |
| Au | Au | 11 | 0 | s_d | 6 |
| B | B | 3 | 0 | s_p | 4 |
| Ba | Ba_sv | 2 | 8 | s_d | 6 |
| Be | Be_sv | 2 | 2 | s_p | 4 |
| Bi | Bi | 5 | 0 | s_p | 4 |
| Br | Br | 7 | 0 | s_p | 4 |
| C | C | 4 | 0 | s_p | 4 |
| Ca | Ca_sv | 2 | 8 | s_d | 6 |
| Cd | Cd | 2 | 10 | s_d | 6 |
| Ce | Ce | 4 | 8 | f_d_s | 13 |
| Cl | Cl | 5 | 2 | p | 3 |
| Co | Co | 9 | 0 | s_d | 6 |
| Cr | Cr_pv | 6 | 6 | s_d | 6 |
| Cs | Cs_sv | 1 | 8 | s_d | 6 |
| Cu | Cu_pv | 11 | 6 | s_d | 6 |
| Dy | Dy_3 | 12 | 3 | s_f | 8 |
| Er | Er_3 | 14 | 5 | f_s | 8 |
| Eu | Eu | 9 | 8 | f_s | 8 |
| F | F | 5 | 2 | p | 3 |
| Fe | Fe_pv | 8 | 6 | s_d | 6 |
| Ga | Ga_d | 3 | 10 | s_p | 4 |
| Gd | Gd | 10 | 8 | f_d_s | 8 |
| Ge | Ge_d | 4 | 10 | s_p | 4 |
| H | H | 1 | 0 | s | 1 |
| He | He | 2 | 0 | s | 1 |
| Hf | Hf_pv | 4 | 6 | s_d | 6 |
| Hg | Hg | 12 | 0 | s_p_d | 9 |
| I | I | 7 | 0 | s_p | 4 |
| In | In_d | 3 | 10 | s_p | 4 |
| Ir | Ir | 9 | 0 | s_d | 6 |
| K | K_sv | 1 | 8 | s_d | 6 |
| Kr | Kr | 8 | 0 | s_p | 4 |
| La | La | 3 | 8 | s_d_f | 13 |
| Li | Li_sv | 1 | 2 | s | 1 |
| Lu | Lu_3 | 17 | 8 | f_d_s | 13 |
| Mg | Mg_pv | 2 | 6 | s_p | 4 |
| Mn | Mn_pv | 7 | 6 | s_d | 6 |
| Mo | Mo_pv | 6 | 6 | s_d | 6 |
| N | N | 3 | 2 | p | 3 |
| Na | Na_pv | 1 | 6 | s_p | 4 |
| Nb | Nb_pv | 5 | 6 | s_d | 6 |
| Nd | Nd_3 | 6 | 5 | f_s | 8 |
| Ne | Ne | 8 | 0 | s_p | 4 |
| Ni | Ni_pv | 10 | 6 | s_d | 6 |
| O | O | 4 | 2 | p | 3 |
| Os | Os_pv | 8 | 6 | s_d | 6 |
| P | P | 5 | 0 | s_p | 4 |
| Pb | Pb_d | 4 | 10 | s_p | 4 |
| Pd | Pd | 10 | 0 | s_d | 6 |
| Pt | Pt | 10 | 0 | s_d | 6 |
| Rb | Rb_sv | 1 | 8 | s_d | 6 |
| Re | Re_pv | 7 | 6 | s_d | 6 |
| Rh | Rh_pv | 9 | 6 | s_d | 6 |
| Ru | Ru_pv | 8 | 6 | s_d | 6 |
| S | S | 4 | 2 | p | 3 |
| Sb | Sb | 5 | 0 | s_p | 4 |
| Sc | Sc_sv | 3 | 8 | s_d | 6 |
| Se | Se | 6 | 0 | s_p | 4 |
| Si | Si | 4 | 0 | s_p | 4 |
| Sm | Sm_3 | 8 | 3 | f_s | 8 |
| Sn | Sn_d | 4 | 10 | s_p | 4 |
| Sr | Sr_sv | 2 | 8 | s_d | 6 |
| Ta | Ta_pv | 5 | 6 | s_d | 6 |
| Tb | Tb_3 | 9 | 0 | f_s | 8 |
| Tc | Tc_pv | 7 | 6 | s_d | 6 |
| Te | Te | 6 | 0 | s_p | 4 |
| Th | Th | 4 | 8 | d_s | 6 |
| Ti | Ti_pv | 4 | 6 | s_d | 6 |
| Tl | Tl_d | 3 | 10 | s_p | 4 |
| U | U | 9 | 5 | f_s | 8 |
| V | V_pv | 5 | 6 | s_d | 6 |
| W | W_pv | 6 | 0 | s_d | 6 |
| Xe | Xe | 8 | 0 | s_p | 4 |
| Y | Y_sv | 3 | 8 | s_d | 6 |
| Zn | Zn | 12 | 0 | s_p_d | 9 |
| Zr | Zr_sv | 4 | 8 | s_d | 6 |

Table S2: MaxDiffs of all the materials under investigation for k-grid on which Wannierization was done and K-points on high symmetry k-points in the Brillouin zone.

| JID | Formula | Maxdiff_k-grid  (eV) | Maxdiff_HighSymBZ  (eV) |
| --- | --- | --- | --- |
| JVASP-22723 | MgMoN2 | 4.32E-05 | 0.017405 |
| JVASP-5296 | CaH2 | 4.33E-05 | 0.053173 |
| JVASP-35664 | MgBe2P2 | 4.50E-05 | 0.018599 |
| JVASP-5284 | SrH2 | 4.50E-05 | 0.023173 |
| JVASP-37905 | BAs | 4.54E-05 | 0.367839 |
| JVASP-35948 | Sc2ZnSe4 | 4.83E-05 | 0.059923 |
| JVASP-18522 | Ba3PbO | 4.85E-05 | 0.522277 |
| JVASP-20306 | CoAs3 | 4.90E-05 | 0.086862 |
| JVASP-35673 | Li2MgSi | 4.90E-05 | 0.086105 |
| JVASP-38129 | Sr6Sn2NF | 4.92E-05 | 0.042597 |
| JVASP-6343 | KAuSe | 4.94E-05 | 0.009004 |
| JVASP-31349 | As | 4.95E-05 | 0.011968 |
| JVASP-15490 | Cu2InLu | 4.97E-05 | 0.097636 |
| JVASP-18632 | SrCuBi | 4.97E-05 | 0.314764 |
| JVASP-17656 | Ca3PbO | 4.98E-05 | 0.121857 |
| JVASP-18650 | Na2CdSn | 5.04E-05 | 0.082132 |
| JVASP-21051 | Ag3AsO4 | 5.04E-05 | 0.173215 |
| JVASP-39881 | Li2CaSn | 5.05E-05 | 0.096495 |
| JVASP-6436 | Al2Te3 | 5.05E-05 | 0.015459 |
| JVASP-21894 | NaCdSb | 5.06E-05 | 0.094044 |
| JVASP-34818 | N6O2NiPb2 | 5.06E-05 | 0.225216 |
| JVASP-35072 | SmSbTe | 5.07E-05 | 0.14186 |
| JVASP-36192 | BC2N | 5.07E-05 | 0.004877 |
| JVASP-35680 | PbS | 5.08E-05 | 0.178845 |
| JVASP-36475 | Sr3As2 | 5.08E-05 | 0.871542 |
| JVASP-20571 | PIr2 | 5.09E-05 | 0.277482 |
| JVASP-48 | C | 5.09E-05 | 0.098172 |
| JVASP-17375 | CaSbAu | 5.10E-05 | 0.16777 |
| JVASP-34485 | ZrCoF6 | 5.10E-05 | 0.0748 |
| JVASP-22726 | Na2TlSb | 5.11E-05 | 0.180916 |
| JVASP-35715 | ErH3 | 5.11E-05 | 0.313914 |
| JVASP-6367 | RbAuSe | 5.11E-05 | 1.332267 |
| JVASP-40350 | LiZn2Pt | 5.12E-05 | 0.177473 |
| JVASP-17310 | Nb4C3 | 5.13E-05 | 0.094808 |
| JVASP-36204 | BP | 5.13E-05 | 0.094925 |
| JVASP-42020 | Sr2PtAu | 5.13E-05 | 0.123239 |
| JVASP-17560 | Sr3PbO | 5.14E-05 | 0.295131 |
| JVASP-36617 | CdSiO3 | 5.14E-05 | 0.022043 |
| JVASP-29442 | Bi4Te3S8 | 5.15E-05 | 0.114513 |
| JVASP-37755 | Na3Tl | 5.15E-05 | 0.206247 |
| JVASP-8091 | CaAgAs | 5.15E-05 | 0.089704 |
| JVASP-15705 | SrAgAs | 5.16E-05 | 0.438269 |
| JVASP-21611 | B4ScOs3 | 5.16E-05 | 0.056589 |
| JVASP-27330 | Rb2Na4Co2O5 | 5.16E-05 | 0.059129 |
| JVASP-39985 | MgSiRh2 | 5.16E-05 | 0.210365 |
| JVASP-18848 | DySnAu | 5.17E-05 | 0.040716 |
| JVASP-22031 | O2TeTl6 | 5.17E-05 | 0.017705 |
| JVASP-26630 | Bi2Rh2O7 | 5.17E-05 | 0.612321 |
| JVASP-40227 | Ca2AsAu | 5.17E-05 | 0.057846 |
| JVASP-54575 | YInAu | 5.17E-05 | 0.574582 |
| JVASP-54890 | RbW3Cl9 | 5.17E-05 | 0.052415 |
| JVASP-60262 | Bi2O3 | 5.17E-05 | 0.002217 |
| JVASP-36486 | Sr3BiSb | 5.18E-05 | 1.151404 |
| JVASP-20244 | Sb3Dy4 | 5.19E-05 | 0.09582 |
| JVASP-39117 | Sc2GaIr | 5.19E-05 | 0.043956 |
| JVASP-56232 | LiCaBi | 5.19E-05 | 0.026433 |
| JVASP-59656 | InAs | 5.19E-05 | 0.105009 |
| JVASP-18469 | Na3Bi | 5.20E-05 | 0.15508 |
| JVASP-60512 | As2S3 | 5.20E-05 | 0.00064 |
| JVASP-20054 | PS | 5.21E-05 | 0.001141 |
| JVASP-6316 | PPdSe | 5.21E-05 | 0.017536 |
| JVASP-22931 | Ca5Sb3 | 5.22E-05 | 0.048487 |
| JVASP-828 | B | 5.22E-05 | 0.120179 |
| JVASP-27847 | SiS | 5.22E-05 | 0.025219 |
| JVASP-16134 | NaTeAu | 5.23E-05 | 0.06442 |
| JVASP-19151 | YCu3Sb4O2 | 5.24E-05 | 0.040541 |
| JVASP-13733 | TlPt | 5.25E-05 | 0.250306 |
| JVASP-23116 | TbGa3Os | 5.25E-05 | 0.076779 |
| JVASP-39802 | Li2AgHg | 5.25E-05 | 0.225189 |
| JVASP-59757 | YMn6Sn6 | 5.25E-05 | 0.065648 |
| JVASP-20042 | W6CCl8 | 5.25E-05 | 0.010646 |
| JVASP-20075 | TiIr3 | 5.26E-05 | 0.11405 |
| JVASP-21401 | O6NaSr3Ir | 5.26E-05 | 0.045737 |
| JVASP-41948 | Rb2Zr2O5 | 5.26E-05 | 0.003785 |
| JVASP-46728 | Li4Mn3SbP4O6 | 5.26E-05 | 0.011278 |
| JVASP-21694 | HfPd3 | 5.27E-05 | 0.190175 |
| JVASP-38127 | Ca6Sn2NF | 5.27E-05 | 0.079528 |
| JVASP-39859 | Li2MgSn | 5.27E-05 | 0.141151 |
| JVASP-28006 | ThTe2I2 | 5.27E-05 | 0.000556 |
| JVASP-16775 | HfBeSi | 5.28E-05 | 0.083847 |
| JVASP-18737 | Li3Pb | 5.28E-05 | 0.225803 |
| JVASP-23869 | S2Pd3Bi2 | 5.28E-05 | 0.213285 |
| JVASP-38781 | ZnPd2Au | 5.28E-05 | 0.085314 |
| JVASP-40246 | Ba2Sc2O5 | 5.28E-05 | 0.003424 |
| JVASP-42040 | LiInPt2 | 5.28E-05 | 0.167202 |
| JVASP-25192 | S | 5.29E-05 | 0.012836 |
| JVASP-26567 | Bi2Os2O7 | 5.29E-05 | 0.200211 |
| JVASP-26681 | Na3OsO5 | 5.29E-05 | 0.145601 |
| JVASP-35449 | AlPPt5 | 5.29E-05 | 0.088564 |
| JVASP-7855 | SSn | 5.29E-05 | 0.050631 |
| JVASP-10484 | O6SrBa2Ir | 5.30E-05 | 0.069061 |
| JVASP-12781 | ZrAs2 | 5.30E-05 | 0.056552 |
| JVASP-15725 | RbTeAu | 5.30E-05 | 0.01413 |
| JVASP-22657 | LiMgBi | 5.30E-05 | 0.247189 |
| JVASP-36456 | Sr3AsN | 5.30E-05 | 0.562953 |
| JVASP-840 | Br | 5.30E-05 | 0.042014 |
| JVASP-60235 | P | 5.30E-05 | 0.031239 |
| JVASP-18521 | Ca3SnO | 5.31E-05 | 0.046223 |
| JVASP-22758 | Al7DyAu3 | 5.31E-05 | 0.148478 |
| JVASP-26224 | AuIn2Na3 | 5.31E-05 | 0.068179 |
| JVASP-40327 | LaCdAg2 | 5.31E-05 | 1.12534 |
| JVASP-42099 | ThGaAu2 | 5.31E-05 | 0.0728 |
| JVASP-56172 | Mg3Si8Ir3 | 5.31E-05 | 0.341147 |
| JVASP-60596 | PdS6Ta2 | 5.31E-05 | 0.586668 |
| JVASP-6391 | Al3Te3I | 5.31E-05 | 0.020772 |
| JVASP-21502 | Li2RhF6 | 5.32E-05 | 0.068223 |
| JVASP-22441 | O6Na3Cd2Ir | 5.32E-05 | 0.0421 |
| JVASP-25037 | Bi2Ir2O7 | 5.32E-05 | 0.191255 |
| JVASP-54631 | CaCdPb | 5.32E-05 | 0.159956 |
| JVASP-21389 | O6CuSr3Pt | 5.33E-05 | 0.105104 |
| JVASP-23848 | SrRu4Sb2 | 5.33E-05 | 0.085358 |
| JVASP-46057 | Li2BiO3 | 5.33E-05 | 0.009114 |
| JVASP-60242 | AsSi | 5.33E-05 | 0.223623 |
| JVASP-12547 | OKBa4Bi3 | 5.34E-05 | 0.047526 |
| JVASP-16409 | K2IrF6 | 5.34E-05 | 0.200698 |
| JVASP-36449 | Sr3BiP | 5.34E-05 | 0.866602 |
| JVASP-36506 | Ba3BiAs | 5.34E-05 | 0.636969 |
| JVASP-39971 | MgTaRh2 | 5.34E-05 | 0.091662 |
| JVASP-41358 | HfBeRh2 | 5.34E-05 | 0.044098 |
| JVASP-17289 | LiYGe | 5.35E-05 | 0.025965 |
| JVASP-21459 | Nb3CoS6 | 5.35E-05 | 0.091913 |
| JVASP-36658 | YN | 5.35E-05 | 0.075493 |
| JVASP-37063 | TcN | 5.35E-05 | 0.094244 |
| JVASP-6340 | RbAuS | 5.35E-05 | 0.010753 |
| JVASP-15227 | Ge2Sb2Te5 | 5.36E-05 | 0.10685 |
| JVASP-17400 | Li2GeHg | 5.36E-05 | 0.098287 |
| JVASP-30934 | Ba4Bi3 | 5.36E-05 | 0.19341 |
| JVASP-35095 | TcB2 | 5.36E-05 | 0.023502 |
| JVASP-36794 | TlAuO2 | 5.36E-05 | 0.201095 |
| JVASP-40778 | SrTePd | 5.36E-05 | 0.11811 |
| JVASP-5743 | Nb3Cl8 | 5.36E-05 | 0.008838 |
| JVASP-16583 | LiAl2Ir | 5.37E-05 | 0.216081 |
| JVASP-26528 | Rb2IrF6 | 5.37E-05 | 0.202426 |
| JVASP-31211 | MgTa2N3 | 5.37E-05 | 0.115378 |
| JVASP-35832 | Ca2H3Br | 5.37E-05 | 0.005594 |
| JVASP-40057 | Li2NdTl | 5.37E-05 | 1.225145 |
| JVASP-56252 | Ta3AlC2 | 5.37E-05 | 0.072709 |
| JVASP-56367 | K3Bi | 5.37E-05 | 0.202309 |
| JVASP-59088 | Rb3Os2Br9 | 5.37E-05 | 0.104901 |
| JVASP-20014 | YCl3 | 5.37E-05 | 0.001752 |
| JVASP-6265 | AlPS4 | 5.37E-05 | 0.030988 |
| JVASP-1071 | Sb2Te3 | 5.38E-05 | 0.110708 |
| JVASP-19712 | LiCd | 5.38E-05 | 0.286685 |
| JVASP-38968 | TaBeO3 | 5.38E-05 | 0.15217 |
| JVASP-41716 | LiSbRh2 | 5.38E-05 | 0.19818 |
| JVASP-60264 | TbCl3 | 5.38E-05 | 1.240384 |
| JVASP-12980 | BiTePd | 5.39E-05 | 0.081684 |
| JVASP-35656 | HfBRh3 | 5.39E-05 | 0.054821 |
| JVASP-36484 | Sr3Sb2 | 5.39E-05 | 0.943499 |
| JVASP-38695 | MgPdAu2 | 5.39E-05 | 0.232254 |
| JVASP-12137 | Bi2Pt | 5.40E-05 | 0.393971 |
| JVASP-281 | SnO | 5.40E-05 | 0.023182 |
| JVASP-38827 | ZrAlRu2 | 5.40E-05 | 0.053349 |
| JVASP-9955 | ZnMo2O4 | 5.40E-05 | 0.022895 |
| JVASP-10740 | TiS2 | 5.41E-05 | 0.014572 |
| JVASP-20861 | CaPd3O4 | 5.41E-05 | 0.089427 |
| JVASP-36476 | Ba3As2 | 5.41E-05 | 0.951466 |
| JVASP-40025 | TiBeRh2 | 5.41E-05 | 0.081762 |
| JVASP-40240 | ScCdAg2 | 5.41E-05 | 0.045617 |
| JVASP-40354 | LiZn2Au | 5.41E-05 | 0.156521 |
| JVASP-4501 | P2VSe6Ag | 5.41E-05 | 0.028144 |
| JVASP-45852 | Li2Nb2Fe3O0 | 5.41E-05 | 0.089186 |
| JVASP-55027 | HfAlPt | 5.41E-05 | 0.06965 |
| JVASP-16868 | Te4Hf5 | 5.42E-05 | 0.096368 |
| JVASP-21379 | NaLuPd6O8 | 5.42E-05 | 0.098971 |
| JVASP-22724 | NaLi2Bi | 5.42E-05 | 0.297618 |
| JVASP-36622 | CaGeO3 | 5.42E-05 | 0.002926 |
| JVASP-41892 | TaAlOs2 | 5.42E-05 | 0.116019 |
| JVASP-43095 | CrSbO4 | 5.42E-05 | 0.058823 |
| JVASP-8660 | SnI6Cs2 | 5.42E-05 | 0.142955 |
| JVASP-6328 | KAuS | 5.42E-05 | 0.012649 |
| JVASP-13951 | BaAgBi | 5.43E-05 | 0.289616 |
| JVASP-1492 | YSbPt | 5.43E-05 | 0.081632 |
| JVASP-15344 | NaZnSb | 5.43E-05 | 0.074188 |
| JVASP-17368 | Hf3Cu4Si2 | 5.43E-05 | 0.084492 |
| JVASP-35817 | TiSnO3 | 5.43E-05 | 0.006272 |
| JVASP-39126 | ScAlCO | 5.43E-05 | 0.01186 |
| JVASP-44507 | Li2MnBAsO7 | 5.43E-05 | 0.026508 |
| JVASP-89 | Bi2Te3 | 5.43E-05 | 0.094955 |
| JVASP-922 | Mn | 5.43E-05 | 0.086366 |
| JVASP-13544 | As2Se3 | 5.43E-05 | 0.000401 |
| JVASP-27848 | TbCBr | 5.43E-05 | 0.465161 |
| JVASP-14900 | NiMoP | 5.44E-05 | 0.029821 |
| JVASP-16379 | ZrAlPt2 | 5.44E-05 | 0.086316 |
| JVASP-27060 | Hf5Sn4 | 5.44E-05 | 0.097584 |
| JVASP-35748 | AlSb | 5.44E-05 | 0.045298 |
| JVASP-37838 | Ca2AgAu | 5.44E-05 | 0.185809 |
| JVASP-17653 | LiIn2Pt | 5.45E-05 | 0.189208 |
| JVASP-21695 | HfPt3 | 5.45E-05 | 0.103326 |
| JVASP-36473 | Ba3SbN | 5.45E-05 | 0.102073 |
| JVASP-38839 | HgPd2Au | 5.45E-05 | 0.099914 |
| JVASP-41755 | LiGaCu2 | 5.45E-05 | 0.221399 |
| JVASP-949 | O | 5.45E-05 | 0.012494 |
| JVASP-6313 | AlSiTe3 | 5.45E-05 | 0.02758 |
| JVASP-14201 | Ta2InC | 5.46E-05 | 0.066648 |
| JVASP-15356 | SmSnAu | 5.46E-05 | 0.050005 |
| JVASP-32759 | BiBr | 5.46E-05 | 0.005752 |
| JVASP-36500 | Ba3AsN | 5.46E-05 | 0.256379 |
| JVASP-37308 | SrGaSnH | 5.46E-05 | 0.028195 |
| JVASP-5575 | Nb2Te6I | 5.46E-05 | 0.008052 |
| JVASP-8751 | Sb2Te4Pb | 5.46E-05 | 0.060487 |
| JVASP-13955 | Se2TeBi2 | 5.47E-05 | 0.040092 |
| JVASP-36505 | Ba3SbAs | 5.47E-05 | 0.614676 |
| JVASP-18345 | BaBi3 | 5.48E-05 | 0.203877 |
| JVASP-18785 | ZnPt3 | 5.48E-05 | 0.318563 |
| JVASP-26325 | O3SbAg | 5.48E-05 | 0.074631 |
| JVASP-35659 | LaPd3C | 5.48E-05 | 0.034075 |
| JVASP-36474 | Ca3BiP | 5.48E-05 | 1.617637 |
| JVASP-36512 | Ca3Bi2 | 5.48E-05 | 0.279079 |
| JVASP-50253 | Tb2SbO2 | 5.48E-05 | 0.042995 |
| JVASP-52847 | Ag7NO6 | 5.48E-05 | 0.067049 |
| JVASP-5410 | BiSe | 5.48E-05 | 0.137126 |
| JVASP-81597 | LiPbAu2 | 5.48E-05 | 0.00233 |
| JVASP-19513 | P4S3 | 5.48E-05 | 0.000517 |
| JVASP-16264 | Y3GaC | 5.49E-05 | 0.809369 |
| JVASP-17331 | LiNdGe | 5.49E-05 | 0.06515 |
| JVASP-20769 | OsSe2 | 5.49E-05 | 0.105899 |
| JVASP-23691 | Nb3PbS6 | 5.49E-05 | 0.174409 |
| JVASP-30451 | Li2DyTl | 5.49E-05 | 1.240427 |
| JVASP-37928 | CaTl2Cd | 5.49E-05 | 0.716654 |
| JVASP-40488 | Nb3GeS6 | 5.49E-05 | 0.006944 |
| JVASP-593 | TaS2 | 5.49E-05 | 0.041688 |
| JVASP-7632 | CdSnSb2 | 5.49E-05 | 0.0408 |
| JVASP-7822 | CaCdGe | 5.49E-05 | 0.118116 |
| JVASP-8659 | SnRb2I6 | 5.49E-05 | 0.176222 |
| JVASP-15470 | Hf2SnC | 5.50E-05 | 0.075177 |
| JVASP-21665 | Si4Ca3Ir4 | 5.50E-05 | 0.102265 |
| JVASP-36709 | YWN3 | 5.50E-05 | 0.005058 |
| JVASP-39816 | Li2CaPb | 5.50E-05 | 0.193062 |
| JVASP-40455 | ThCdAg2 | 5.50E-05 | 0.698539 |
| JVASP-41154 | Na2TlBi | 5.50E-05 | 0.370648 |
| JVASP-41314 | NaNdHg2 | 5.50E-05 | 0.15715 |
| JVASP-41779 | LiPd3 | 5.50E-05 | 0.33928 |
| JVASP-9080 | Te5Hf | 5.50E-05 | 0.046801 |
| JVASP-13043 | GeBi4Te7 | 5.51E-05 | 0.130166 |
| JVASP-16648 | Sc6Te2Os | 5.51E-05 | 0.13592 |
| JVASP-18539 | Sb3Au | 5.51E-05 | 0.197057 |
| JVASP-5314 | UCl5 | 5.51E-05 | 0.044454 |
| JVASP-54836 | TaCr2 | 5.51E-05 | 0.055608 |
| JVASP-60482 | Cl3Ti | 5.51E-05 | 0.022204 |
| JVASP-60597 | PdSe6Ta2 | 5.51E-05 | 0.574606 |
| JVASP-6331 | PPdS | 5.51E-05 | 0.008439 |
| JVASP-15830 | WS2 | 5.52E-05 | 0.011584 |
| JVASP-16405 | SmMgGa | 5.52E-05 | 0.060605 |
| JVASP-17430 | Cu2ZnSe4Sn | 5.52E-05 | 0.018753 |
| JVASP-21041 | CaMgSn | 5.52E-05 | 0.058078 |
| JVASP-21995 | BaZn2Sb2 | 5.52E-05 | 0.038431 |
| JVASP-38414 | Li2TlSn | 5.52E-05 | 0.145508 |
| JVASP-38528 | NaCa2In | 5.52E-05 | 0.192563 |
| JVASP-41381 | MgGaIr2 | 5.52E-05 | 0.281334 |
| JVASP-41773 | LiPdAu2 | 5.52E-05 | 0.200072 |
| JVASP-60525 | AgP2Se6V | 5.52E-05 | 0.008512 |
| JVASP-6190 | BPS4 | 5.52E-05 | 0.043924 |
| JVASP-21571 | ScB4Ir3 | 5.53E-05 | 0.197459 |
| JVASP-22384 | Ta3SnS6 | 5.53E-05 | 0.04876 |
| JVASP-26103 | TbRh3C | 5.53E-05 | 0.106773 |
| JVASP-38820 | Ta2CrRu | 5.53E-05 | 0.106697 |
| JVASP-38967 | TiPt3 | 5.53E-05 | 0.064783 |
| JVASP-40093 | HfSnRh2 | 5.53E-05 | 0.050765 |
| JVASP-40476 | LiCuS | 5.53E-05 | 0.055437 |
| JVASP-4894 | TaTe4Ir | 5.53E-05 | 0.11078 |
| JVASP-8366 | Ca3SbN | 5.53E-05 | 0.871341 |
| JVASP-12648 | B2O6MnSn | 5.54E-05 | 0.141199 |
| JVASP-20406 | Te2Os | 5.54E-05 | 0.089276 |
| JVASP-35524 | InSiPt5 | 5.54E-05 | 0.099569 |
| JVASP-37675 | NaPd2Pb | 5.54E-05 | 0.129641 |
| JVASP-3909 | SnSb2Te4 | 5.54E-05 | 0.082571 |
| JVASP-40022 | TiBe2Ir | 5.54E-05 | 0.137928 |
| JVASP-41897 | LiErTl2 | 5.54E-05 | 0.098012 |
| JVASP-44705 | MnSb4O2 | 5.54E-05 | 0.137984 |
| JVASP-8441 | PbO2 | 5.54E-05 | 0.024315 |
| JVASP-60308 | SbAsO3 | 5.54E-05 | 0.004065 |
| JVASP-60612 | As2Si | 5.54E-05 | 0.026591 |
| JVASP-13835 | YN | 5.55E-05 | 0.011477 |
| JVASP-15028 | NdPt2 | 5.55E-05 | 0.259047 |
| JVASP-15807 | SrZn2Sb2 | 5.55E-05 | 0.01275 |
| JVASP-18066 | ThPb3 | 5.55E-05 | 0.27339 |
| JVASP-18176 | Rb2RhF6 | 5.55E-05 | 0.152128 |
| JVASP-18818 | CaZnSn | 5.55E-05 | 0.021558 |
| JVASP-25104 | Cl | 5.55E-05 | 0.023848 |
| JVASP-36672 | Bi2O3 | 5.55E-05 | 0.051108 |
| JVASP-40398 | Rb2Hf2O5 | 5.55E-05 | 0.004691 |
| JVASP-31418 | SbAsO4 | 5.55E-05 | 0.002056 |
| JVASP-18118 | YMgIn | 5.56E-05 | 0.03886 |
| JVASP-18251 | S2Ni3Pb2 | 5.56E-05 | 0.07287 |
| JVASP-3477 | Nd3InN | 5.56E-05 | 1.062091 |
| JVASP-40262 | Sr2CdSn | 5.56E-05 | 0.031129 |
| JVASP-54439 | FeB2W2 | 5.56E-05 | 0.102174 |
| JVASP-81 | GaSe | 5.56E-05 | 0.022332 |
| JVASP-16693 | SmMgTl | 5.57E-05 | 0.117037 |
| JVASP-18603 | InErAu2 | 5.57E-05 | 0.497221 |
| JVASP-35414 | ZnPd5Se | 5.57E-05 | 0.068456 |
| JVASP-35654 | Hf2SN2 | 5.57E-05 | 0.017404 |
| JVASP-38462 | NaLi2Pb | 5.57E-05 | 0.218241 |
| JVASP-40303 | LiGaPd2 | 5.57E-05 | 0.189129 |
| JVASP-41312 | NaNdAu2 | 5.57E-05 | 0.689227 |
| JVASP-54354 | Hf5CuPb3 | 5.57E-05 | 0.078122 |
| JVASP-6016 | SiS2 | 5.57E-05 | 0.017498 |
| JVASP-6520 | P4Se5 | 5.57E-05 | 0.001476 |
| JVASP-15826 | LiMgSnAu | 5.58E-05 | 0.142013 |
| JVASP-17435 | SmMgPt | 5.58E-05 | 0.205206 |
| JVASP-18948 | SnPd3 | 5.58E-05 | 0.216293 |
| JVASP-21887 | Ta3B2 | 5.58E-05 | 0.08935 |
| JVASP-34824 | O2Bi | 5.58E-05 | 0.023303 |
| JVASP-38028 | SbOF | 5.58E-05 | 0.003736 |
| JVASP-39934 | LiNdTl2 | 5.58E-05 | 0.26656 |
| JVASP-41324 | MgGaRh2 | 5.58E-05 | 0.356307 |
| JVASP-6451 | As2O3 | 5.58E-05 | 0.014042 |
| JVASP-17747 | CaPb3 | 5.59E-05 | 0.255041 |
| JVASP-20225 | TbTl3 | 5.59E-05 | 0.43425 |
| JVASP-21492 | SrPd3O4 | 5.59E-05 | 0.08309 |
| JVASP-21564 | HfSiPd | 5.59E-05 | 0.090112 |
| JVASP-23794 | HfFeP | 5.59E-05 | 0.087341 |
| JVASP-35401 | Rb2PdCl4 | 5.59E-05 | 0.078061 |
| JVASP-36479 | Ba3BiAs | 5.59E-05 | 0.940214 |
| JVASP-37414 | TaW3 | 5.59E-05 | 0.146552 |
| JVASP-40599 | Sm2MgAl | 5.59E-05 | 0.912603 |
| JVASP-51117 | InP2Pb | 5.59E-05 | 0.098135 |
| JVASP-9160 | Sb4Th3 | 5.59E-05 | 0.073947 |
| JVASP-13536 | SbTe | 5.59E-05 | 0.026586 |
| JVASP-13856 | GaSe | 5.60E-05 | 0.019072 |
| JVASP-17936 | Pd3PbC | 5.60E-05 | 0.232942 |
| JVASP-35490 | TlSiPt5 | 5.60E-05 | 0.094142 |
| JVASP-35597 | LiNbSe2 | 5.60E-05 | 0.007773 |
| JVASP-36454 | Sr3SbP | 5.60E-05 | 0.682364 |
| JVASP-36480 | Sr3BiAs | 5.60E-05 | 1.337981 |
| JVASP-41890 | Sc2AgOs | 5.60E-05 | 0.124075 |
| JVASP-5962 | SiP2 | 5.60E-05 | 0.020855 |
| JVASP-22653 | Sr3BiN | 5.61E-05 | 0.905619 |
| JVASP-23550 | BaPPt | 5.61E-05 | 0.078295 |
| JVASP-37579 | Zn2CuIr | 5.61E-05 | 0.258814 |
| JVASP-4636 | MgAl2Se4 | 5.61E-05 | 0.017069 |
| JVASP-143 | GaS | 5.62E-05 | 0.003518 |
| JVASP-15801 | KHgAs | 5.62E-05 | 0.024817 |
| JVASP-16879 | ZrGaAu | 5.62E-05 | 0.051511 |
| JVASP-20172 | Pt3O4 | 5.62E-05 | 0.162819 |
| JVASP-29951 | Cu2HgI4 | 5.62E-05 | 0.093071 |
| JVASP-35477 | CdAsPt5 | 5.62E-05 | 0.086033 |
| JVASP-35833 | CaBiAu | 5.62E-05 | 0.11491 |
| JVASP-38141 | YWN3 | 5.62E-05 | 0.004391 |
| JVASP-39153 | LaH3 | 5.62E-05 | 0.041751 |
| JVASP-40551 | LiHfPt2 | 5.62E-05 | 0.092688 |
| JVASP-40646 | SmPbAu2 | 5.62E-05 | 1.526533 |
| JVASP-41709 | LiNdHg2 | 5.62E-05 | 0.774928 |
| JVASP-56928 | Ba2Bi | 5.62E-05 | 0.134273 |
| JVASP-60301 | PSi | 5.62E-05 | 0.748798 |
| JVASP-6523 | Sb2S3 | 5.62E-05 | 0.000494 |
| JVASP-17537 | Au2Pb | 5.63E-05 | 0.250281 |
| JVASP-18738 | Li7Pb2 | 5.63E-05 | 0.178385 |
| JVASP-18847 | TbSnAu | 5.63E-05 | 0.045861 |
| JVASP-35289 | TbInPt4 | 5.63E-05 | 0.17699 |
| JVASP-35667 | Ni3SnN | 5.63E-05 | 0.171384 |
| JVASP-36466 | Ba3BiN | 5.63E-05 | 0.314954 |
| JVASP-36635 | VHgO3 | 5.63E-05 | 0.048964 |
| JVASP-36637 | MgZrO3 | 5.63E-05 | 0.015217 |
| JVASP-37250 | SmSnPd2 | 5.63E-05 | 0.14505 |
| JVASP-39819 | GaGeRu2 | 5.63E-05 | 0.086274 |
| JVASP-6292 | PPdSe | 5.63E-05 | 0.017061 |
| JVASP-12395 | Ba2LuCu3O6 | 5.64E-05 | 0.085103 |
| JVASP-16039 | As4Ta5 | 5.64E-05 | 0.046814 |
| JVASP-17592 | GePt2 | 5.64E-05 | 0.118665 |
| JVASP-18065 | PtPb | 5.64E-05 | 0.1636 |
| JVASP-2817 | O4K4Ir | 5.64E-05 | 0.050784 |
| JVASP-35222 | P3Pd | 5.64E-05 | 0.044415 |
| JVASP-36625 | ZnSnO3 | 5.64E-05 | 0.011275 |
| JVASP-38357 | InSb3 | 5.64E-05 | 0.064795 |
| JVASP-40248 | ScZnPd2 | 5.64E-05 | 0.076409 |
| JVASP-40282 | SrInHg2 | 5.64E-05 | 0.185516 |
| JVASP-41243 | CaYRh2 | 5.64E-05 | 0.047541 |
| JVASP-931 | Na | 5.64E-05 | 0.06535 |
| JVASP-14698 | Al2Nd | 5.65E-05 | 0.17108 |
| JVASP-15278 | TbBRh3 | 5.65E-05 | 1.057339 |
| JVASP-16418 | Y6FeSb2 | 5.65E-05 | 0.024281 |
| JVASP-17212 | ErSnAu | 5.65E-05 | 0.121832 |
| JVASP-23773 | B2U | 5.65E-05 | 0.232786 |
| JVASP-30507 | AgBiO2 | 5.65E-05 | 0.018148 |
| JVASP-35937 | Li3YBi2 | 5.65E-05 | 0.075811 |
| JVASP-36123 | InSb | 5.65E-05 | 0.14825 |
| JVASP-41894 | Sc2AgRu | 5.65E-05 | 0.069445 |
| JVASP-13968 | TaGe2 | 5.66E-05 | 0.060441 |
| JVASP-15599 | B2Co2Nd | 5.66E-05 | 0.12367 |
| JVASP-21600 | HfSiPt | 5.66E-05 | 0.051433 |
| JVASP-36504 | Ba3BiP | 5.66E-05 | 0.593679 |
| JVASP-36603 | SrIrO3 | 5.66E-05 | 0.300595 |
| JVASP-39223 | Li2MgHg | 5.66E-05 | 0.128923 |
| JVASP-4101 | SeTe2Bi2 | 5.66E-05 | 0.0769 |
| JVASP-41170 | MgZrAu2 | 5.66E-05 | 0.147987 |
| JVASP-41775 | LiZnAu2 | 5.66E-05 | 0.176514 |
| JVASP-8726 | Li5Sn2 | 5.66E-05 | 0.500335 |
| JVASP-1065 | Te3Tl4Pb | 5.67E-05 | 0.00994 |
| JVASP-14006 | Li2NdSb2 | 5.67E-05 | 0.03303 |
| JVASP-16776 | CaPd3C | 5.67E-05 | 0.087656 |
| JVASP-35823 | YPt3C | 5.67E-05 | 0.150786 |
| JVASP-36515 | Ca3SbAs | 5.67E-05 | 1.177964 |
| JVASP-37330 | TbB4Ir4 | 5.67E-05 | 0.072471 |
| JVASP-38655 | K2PtC2 | 5.67E-05 | 0.133154 |
| JVASP-40379 | Be2IrPt | 5.67E-05 | 0.777482 |
| JVASP-41604 | YAgHg2 | 5.67E-05 | 0.127822 |
| JVASP-6370 | ScAgP2S6 | 5.67E-05 | 0.022102 |
| JVASP-14395 | Hf2SnC | 5.68E-05 | 0.075184 |
| JVASP-15265 | ErBRh3 | 5.68E-05 | 0.948608 |
| JVASP-20925 | Ta4AlC3 | 5.68E-05 | 0.070813 |
| JVASP-35469 | SnPPd5 | 5.68E-05 | 0.053761 |
| JVASP-36513 | Ba3Bi2 | 5.68E-05 | 0.756381 |
| JVASP-37476 | DyYCu2 | 5.68E-05 | 0.775258 |
| JVASP-41374 | Hf2FeOs | 5.68E-05 | 0.082859 |
| JVASP-4846 | PtN | 5.68E-05 | 0.175908 |
| JVASP-8367 | Ca3BiN | 5.68E-05 | 0.096522 |
| JVASP-1008 | Sn | 5.69E-05 | 0.112386 |
| JVASP-12060 | CuBrO2 | 5.69E-05 | 0.068016 |
| JVASP-15230 | KTeAu | 5.69E-05 | 0.060711 |
| JVASP-17712 | SnPt3C | 5.69E-05 | 0.158311 |
| JVASP-18426 | AlCl3 | 5.69E-05 | 0.015272 |
| JVASP-18599 | BaAu5 | 5.69E-05 | 0.093897 |
| JVASP-20350 | ZrSb2 | 5.69E-05 | 0.033712 |
| JVASP-22761 | Al7SmAu3 | 5.69E-05 | 0.047204 |
| JVASP-35067 | KMgBi | 5.69E-05 | 0.16259 |
| JVASP-35890 | HfN2 | 5.69E-05 | 0.034356 |
| JVASP-36464 | Ca3P2 | 5.69E-05 | 0.039757 |
| JVASP-39395 | La2ZnAg | 5.69E-05 | 0.903977 |
| JVASP-39947 | AlZnRh2 | 5.69E-05 | 0.292296 |
| JVASP-40299 | Sr2BiAu | 5.69E-05 | 0.107541 |
| JVASP-40568 | SmCdAg2 | 5.69E-05 | 0.730129 |
| JVASP-41256 | HfGaRh2 | 5.69E-05 | 0.085809 |
| JVASP-51319 | BeSbAs2 | 5.69E-05 | 0.056088 |
| JVASP-5311 | HgPS3 | 5.69E-05 | 0.001849 |
| JVASP-17303 | F6RhCd | 5.70E-05 | 0.070818 |
| JVASP-17305 | AsRu | 5.70E-05 | 0.023521 |
| JVASP-18771 | V3Pb | 5.70E-05 | 0.039235 |
| JVASP-23044 | Ni4SrSn2 | 5.70E-05 | 0.190001 |
| JVASP-37007 | YN | 5.70E-05 | 0.021136 |
| JVASP-38859 | ZrScOs2 | 5.70E-05 | 0.110083 |
| JVASP-41938 | ScSbRh2 | 5.70E-05 | 0.240368 |
| JVASP-52645 | TePb | 5.70E-05 | 0.018873 |
| JVASP-8385 | YCoF5 | 5.70E-05 | 0.08219 |
| JVASP-12058 | Si3H | 5.71E-05 | 0.015653 |
| JVASP-12471 | CuBiS2 | 5.71E-05 | 0.272558 |
| JVASP-36499 | Ba3SbN | 5.71E-05 | 0.216736 |
| JVASP-36514 | Ba3SbP | 5.71E-05 | 0.478637 |
| JVASP-37215 | Si2TcOs | 5.71E-05 | 0.136664 |
| JVASP-41327 | NaSr2Tl | 5.71E-05 | 0.133549 |
| JVASP-41918 | Be2PtRh | 5.71E-05 | 0.464787 |
| JVASP-54925 | ZnI2 | 5.71E-05 | 0.119243 |
| JVASP-56178 | Te2Ir | 5.71E-05 | 0.114266 |
| JVASP-7741 | CaAgP | 5.71E-05 | 0.064193 |
| JVASP-7860 | SnTe | 5.71E-05 | 0.143974 |
| JVASP-895 | I | 5.71E-05 | 0.081238 |
| JVASP-28270 | BiS2 | 5.71E-05 | 0.000284 |
| JVASP-20530 | NbSb2 | 5.72E-05 | 0.023503 |
| JVASP-40971 | ErCdPd2 | 5.72E-05 | 1.472355 |
| JVASP-41105 | LiSnRh2 | 5.72E-05 | 0.207151 |
| JVASP-4358 | GaSe | 5.72E-05 | 0.020853 |
| JVASP-51338 | FePbW2 | 5.72E-05 | 0.075489 |
| JVASP-28311 | SbN | 5.72E-05 | 0.004036 |
| JVASP-6271 | AlClO | 5.72E-05 | 0.129042 |
| JVASP-20331 | Sb2Au | 5.73E-05 | 0.04472 |
| JVASP-23502 | ZrFeP | 5.73E-05 | 0.028187 |
| JVASP-28386 | TaSe2 | 5.73E-05 | 0.017342 |
| JVASP-362 | ZrSe3 | 5.73E-05 | 0.011042 |
| JVASP-36463 | Ca3Sb2 | 5.73E-05 | 1.48697 |
| JVASP-36598 | HfHgO3 | 5.73E-05 | 0.039312 |
| JVASP-36668 | CaBe2P2 | 5.73E-05 | 0.021241 |
| JVASP-36755 | LiAuO2 | 5.73E-05 | 0.032453 |
| JVASP-38836 | Nd2ZnIr | 5.73E-05 | 0.116622 |
| JVASP-38849 | CaBiPd2 | 5.73E-05 | 0.174237 |
| JVASP-40381 | Tb2IrRh | 5.73E-05 | 1.647368 |
| JVASP-4053 | BaMg2Bi2 | 5.73E-05 | 0.052806 |
| JVASP-41701 | ThCdRh2 | 5.73E-05 | 0.497869 |
| JVASP-41879 | Sc2RuPt | 5.73E-05 | 0.090303 |
| JVASP-43952 | Ta2CrNO5 | 5.73E-05 | 0.120075 |
| JVASP-18613 | InLuAu2 | 5.74E-05 | 0.144814 |
| JVASP-20246 | Nb3Os | 5.74E-05 | 0.655549 |
| JVASP-29355 | Ta2Te3 | 5.74E-05 | 0.014695 |
| JVASP-35666 | ScBPd3 | 5.74E-05 | 0.270532 |
| JVASP-36510 | Ca3BiSb | 5.74E-05 | 1.412332 |
| JVASP-36660 | ZnO | 5.74E-05 | 0.020594 |
| JVASP-37780 | Ga3Bi | 5.74E-05 | 0.169646 |
| JVASP-41218 | Mg2AgIr | 5.74E-05 | 0.216045 |
| JVASP-41508 | DyTaRu2 | 5.74E-05 | 1.708933 |
| JVASP-5839 | AlSiTe3 | 5.74E-05 | 0.105368 |
| JVASP-85013 | Fe3Sn2 | 5.74E-05 | 0.300003 |
| JVASP-12475 | Bi4Te7Pb | 5.75E-05 | 0.121728 |
| JVASP-16001 | K2RhF6 | 5.75E-05 | 0.143937 |
| JVASP-17488 | YInPt | 5.75E-05 | 0.110928 |
| JVASP-18777 | ScSnPt2 | 5.75E-05 | 0.090122 |
| JVASP-18806 | Sc6Te2Rh | 5.75E-05 | 0.066057 |
| JVASP-23777 | Hf5Ga3 | 5.75E-05 | 0.071592 |
| JVASP-36462 | Ca3BiAs | 5.75E-05 | 1.582522 |
| JVASP-36760 | KAgO2 | 5.75E-05 | 0.051016 |
| JVASP-39996 | Ba2HgPb | 5.75E-05 | 0.099891 |
| JVASP-41380 | MgBe2As2 | 5.75E-05 | 0.009999 |
| JVASP-41898 | LiEr2Al | 5.75E-05 | 0.286833 |
| JVASP-54796 | DySn3 | 5.75E-05 | 0.321454 |
| JVASP-78460 | RbB | 5.75E-05 | 0.007042 |
| JVASP-6352 | ScAgP2Se6 | 5.75E-05 | 0.010759 |
| JVASP-1106 | Ag2Te | 5.76E-05 | 0.048157 |
| JVASP-14603 | As | 5.76E-05 | 0.069779 |
| JVASP-36455 | Sr3PN | 5.76E-05 | 0.768059 |
| JVASP-37006 | YSb | 5.76E-05 | 0.186397 |
| JVASP-38897 | Bi2Rh3S2 | 5.76E-05 | 0.036194 |
| JVASP-41309 | NaMgTl2 | 5.76E-05 | 0.328675 |
| JVASP-41868 | Sc2NiIr | 5.76E-05 | 0.081201 |
| JVASP-7804 | Se | 5.76E-05 | 0.029335 |
| JVASP-1067 | Bi2Se3 | 5.77E-05 | 0.009389 |
| JVASP-16738 | TiPt3 | 5.77E-05 | 0.171617 |
| JVASP-28345 | GaN | 5.77E-05 | 0.011596 |
| JVASP-36448 | Sr3N2 | 5.77E-05 | 0.043133 |
| JVASP-36492 | Sr3BiP | 5.77E-05 | 1.244565 |
| JVASP-36495 | Ca3BiSb | 5.77E-05 | 0.088645 |
| JVASP-36680 | CaWN3 | 5.77E-05 | 0.009045 |
| JVASP-38536 | Hf3Ge2 | 5.77E-05 | 0.212965 |
| JVASP-40107 | Er2PdRu | 5.77E-05 | 0.082832 |
| JVASP-54938 | ThBi2 | 5.77E-05 | 0.209836 |
| JVASP-16265 | Sm3GaC | 5.78E-05 | 0.171995 |
| JVASP-18802 | SrAgBi | 5.78E-05 | 0.321315 |
| JVASP-21716 | As3W2 | 5.78E-05 | 0.019448 |
| JVASP-21807 | HfAl2 | 5.78E-05 | 0.049969 |
| JVASP-35528 | CdAg2I4 | 5.78E-05 | 0.132979 |
| JVASP-38233 | Rb3Ir | 5.78E-05 | 0.782328 |
| JVASP-39808 | Li2SmIn | 5.78E-05 | 0.375331 |
| JVASP-40627 | ScGaPt2 | 5.78E-05 | 0.083862 |
| JVASP-41014 | BaHg2Pb | 5.78E-05 | 0.180173 |
| JVASP-4657 | SGa | 5.78E-05 | 0.031321 |
| JVASP-10117 | NaCdAs | 5.79E-05 | 0.033051 |
| JVASP-16167 | NbSnS2 | 5.79E-05 | 0.115907 |
| JVASP-18228 | YInAu2 | 5.79E-05 | 0.133043 |
| JVASP-40033 | TiAlRu2 | 5.79E-05 | 0.200049 |
| JVASP-40569 | Ca2ZnRh | 5.79E-05 | 0.467588 |
| JVASP-41039 | ErAgHg2 | 5.79E-05 | 0.185166 |
| JVASP-11397 | SrMgGe | 5.80E-05 | 0.048136 |
| JVASP-16419 | Lu6FeSb2 | 5.80E-05 | 0.561727 |
| JVASP-17771 | NdPb3 | 5.80E-05 | 2.262031 |
| JVASP-35338 | TbSe2 | 5.80E-05 | 0.013068 |
| JVASP-38644 | Ni3Pb | 5.80E-05 | 0.11716 |
| JVASP-39970 | MgTaOs2 | 5.80E-05 | 0.088659 |
| JVASP-40651 | NaCoO2 | 5.80E-05 | 0.176438 |
| JVASP-41924 | LiHfIr2 | 5.80E-05 | 0.107808 |
| JVASP-42014 | Sr2TlCd | 5.80E-05 | 0.874292 |
| JVASP-51277 | KBIr2 | 5.80E-05 | 0.191944 |
| JVASP-56734 | YSnAu | 5.80E-05 | 0.079595 |
| JVASP-57644 | Se4Sm3 | 5.80E-05 | 0.056317 |
| JVASP-12508 | Bi4Th3 | 5.81E-05 | 0.175263 |
| JVASP-13042 | GeSb4Te7 | 5.81E-05 | 0.046166 |
| JVASP-14563 | DyPt3 | 5.81E-05 | 1.297204 |
| JVASP-16728 | Si2DyOs2 | 5.81E-05 | 0.063217 |
| JVASP-29222 | TeBi | 5.81E-05 | 0.142941 |
| JVASP-36496 | Sr3BiAs | 5.81E-05 | 1.041158 |
| JVASP-39135 | Sc2OsAu | 5.81E-05 | 0.175921 |
| JVASP-39169 | ScBiPd2 | 5.81E-05 | 0.118598 |
| JVASP-40207 | CaSnS3 | 5.81E-05 | 0.004444 |
| JVASP-58259 | Te2Ir | 5.81E-05 | 0.069039 |
| JVASP-59502 | Ta5Ge3 | 5.81E-05 | 0.10657 |
| JVASP-6454 | As2O3 | 5.81E-05 | 0.007648 |
| JVASP-12178 | Li2WS4 | 5.82E-05 | 0.013584 |
| JVASP-14316 | Ag3Sb | 5.82E-05 | 0.594312 |
| JVASP-17265 | F6BaIr | 5.82E-05 | 0.080241 |
| JVASP-17941 | PbS | 5.82E-05 | 0.191601 |
| JVASP-25337 | Tl | 5.82E-05 | 0.16945 |
| JVASP-35892 | HfTaNO3 | 5.82E-05 | 0.020377 |
| JVASP-40279 | LiGaPt2 | 5.82E-05 | 0.144159 |
| JVASP-40311 | Sr2LiIn | 5.82E-05 | 0.032547 |
| JVASP-40567 | Sm2NiIr | 5.82E-05 | 0.167324 |
| JVASP-834 | Be | 5.82E-05 | 0.302193 |
| JVASP-15011 | Be2Ta | 5.83E-05 | 0.085531 |
| JVASP-16573 | LuPb3 | 5.83E-05 | 1.148754 |
| JVASP-16662 | SrSbAu | 5.83E-05 | 0.1028 |
| JVASP-19710 | MnSi | 5.83E-05 | 0.043061 |
| JVASP-20554 | Be2Nb | 5.83E-05 | 0.301335 |
| JVASP-36451 | Sr3AsP | 5.83E-05 | 0.665147 |
| JVASP-40280 | SrLi2Sn | 5.83E-05 | 0.107504 |
| JVASP-41257 | HfMgIr2 | 5.83E-05 | 0.115892 |
| JVASP-17609 | BaPb | 5.84E-05 | 0.025777 |
| JVASP-17726 | Cu2InLa | 5.84E-05 | 0.08518 |
| JVASP-21680 | Ca3Hg2 | 5.84E-05 | 0.087861 |
| JVASP-25215 | Sb | 5.84E-05 | 0.072037 |
| JVASP-28416 | Te2W | 5.84E-05 | 0.011868 |
| JVASP-34889 | AgBiSe2 | 5.84E-05 | 0.210789 |
| JVASP-35056 | CaSnHg | 5.84E-05 | 0.035119 |
| JVASP-35086 | TaTi2N3 | 5.84E-05 | 0.053046 |
| JVASP-36465 | Ca3SbN | 5.84E-05 | 0.136585 |
| JVASP-37648 | HgBi3 | 5.84E-05 | 0.346182 |
| JVASP-38043 | YWN3 | 5.84E-05 | 0.007314 |
| JVASP-10637 | BiO2 | 5.85E-05 | 0.356907 |
| JVASP-1082 | Mg2Sn | 5.85E-05 | 0.115452 |
| JVASP-16027 | BaCd2Sb2 | 5.85E-05 | 0.033903 |
| JVASP-16436 | Mg2AgSm | 5.85E-05 | 0.105052 |
| JVASP-17193 | PtN | 5.85E-05 | 0.16272 |
| JVASP-36458 | Sr3SbAs | 5.85E-05 | 0.642363 |
| JVASP-4558 | CS2Ta2 | 5.85E-05 | 0.088331 |
| JVASP-5374 | IBi | 5.85E-05 | 0.004687 |
| JVASP-15721 | Sb2Te | 5.86E-05 | 0.045529 |
| JVASP-17316 | Li2CuAs | 5.86E-05 | 0.10751 |
| JVASP-18015 | Ti3InN | 5.86E-05 | 0.021881 |
| JVASP-20384 | PIr2 | 5.86E-05 | 0.277948 |
| JVASP-36511 | Ba3AsN | 5.86E-05 | 0.474508 |
| JVASP-36756 | LiAgO2 | 5.86E-05 | 0.052119 |
| JVASP-37449 | DyYZn2 | 5.86E-05 | 1.086621 |
| JVASP-40065 | TlCdRh2 | 5.86E-05 | 0.066563 |
| JVASP-40257 | CaMgHg2 | 5.86E-05 | 0.15029 |
| JVASP-56062 | ThRh2 | 5.86E-05 | 0.133021 |
| JVASP-6286 | Sc2NCl2 | 5.86E-05 | 0.005809 |
| JVASP-13736 | SrSbAu | 5.87E-05 | 0.102798 |
| JVASP-16478 | Li2CdPb | 5.87E-05 | 0.415669 |
| JVASP-18252 | CaIn2Au | 5.87E-05 | 0.025341 |
| JVASP-23065 | ScSnPt | 5.87E-05 | 0.077272 |
| JVASP-36501 | Ba3BiN | 5.87E-05 | 0.215547 |
| JVASP-37293 | SmY3 | 5.87E-05 | 1.285054 |
| JVASP-39386 | OsN2 | 5.87E-05 | 0.532056 |
| JVASP-39795 | Li2YTl | 5.87E-05 | 0.112541 |
| JVASP-39853 | Cd2PtRh | 5.87E-05 | 0.103956 |
| JVASP-40196 | CaSnS3 | 5.87E-05 | 0.002009 |
| JVASP-8272 | CaSn2N2 | 5.87E-05 | 0.033623 |
| JVASP-16771 | As2Ta | 5.88E-05 | 0.020265 |
| JVASP-28502 | Te2MoW2Se2S2 | 5.88E-05 | 0.01997 |
| JVASP-34934 | HfAs2 | 5.88E-05 | 0.015976 |
| JVASP-38826 | ZrAlRh2 | 5.88E-05 | 0.060436 |
| JVASP-40531 | LiYHg2 | 5.88E-05 | 0.149196 |
| JVASP-5 | TiO2 | 5.88E-05 | 0.002443 |
| JVASP-802 | Hf | 5.88E-05 | 0.070627 |
| JVASP-13124 | Sr2Pb | 5.89E-05 | 0.027785 |
| JVASP-16786 | PTa | 5.89E-05 | 0.096726 |
| JVASP-18322 | NaBi | 5.89E-05 | 0.102498 |
| JVASP-19850 | SeHg | 5.89E-05 | 0.052411 |
| JVASP-20582 | CuAu3 | 5.89E-05 | 0.141178 |
| JVASP-25210 | Te | 5.89E-05 | 0.057854 |
| JVASP-34163 | Si3W5 | 5.89E-05 | 0.02569 |
| JVASP-35741 | CaGaGeH | 5.89E-05 | 0.014748 |
| JVASP-36702 | CaTcN3 | 5.89E-05 | 0.028277 |
| JVASP-38158 | HgF2 | 5.89E-05 | 0.052564 |
| JVASP-40075 | Li2SmTl | 5.89E-05 | 0.114094 |
| JVASP-41569 | TiGaRu2 | 5.89E-05 | 0.039652 |
| JVASP-41702 | Li2PdPb | 5.89E-05 | 0.368501 |
| JVASP-4456 | CS2Nb2 | 5.89E-05 | 0.047397 |
| JVASP-51474 | YHf2Pb | 5.89E-05 | 0.088917 |
| JVASP-7079 | Se4Cu2ZnSn | 5.89E-05 | 0.030365 |
| JVASP-16008 | TaSnO3 | 5.90E-05 | 0.068259 |
| JVASP-16547 | KHg | 5.90E-05 | 0.031549 |
| JVASP-16886 | ScAs | 5.90E-05 | 0.158455 |
| JVASP-18886 | Ag2Te | 5.90E-05 | 0.161949 |
| JVASP-26860 | SrCu2Sb2 | 5.90E-05 | 0.066647 |
| JVASP-35105 | ThTeAs | 5.90E-05 | 0.047031 |
| JVASP-39063 | SmGaAu2 | 5.90E-05 | 1.602021 |
| JVASP-40578 | SmMgAu2 | 5.90E-05 | 0.19023 |
| JVASP-41095 | LiSnIr2 | 5.90E-05 | 0.192588 |
| JVASP-41249 | HfGaRu2 | 5.90E-05 | 0.055883 |
| JVASP-51567 | YSn6Ru4 | 5.90E-05 | 0.025004 |
| JVASP-8202 | As2SrSn2 | 5.90E-05 | 0.022204 |
| JVASP-1029 | Ti | 5.91E-05 | 0.03713 |
| JVASP-16116 | ScSiAu | 5.91E-05 | 0.041226 |
| JVASP-17852 | Pd3Pb | 5.91E-05 | 0.159582 |
| JVASP-18600 | BeAu | 5.91E-05 | 0.072541 |
| JVASP-23796 | LuSiIr | 5.91E-05 | 0.311389 |
| JVASP-30450 | Li2DyIn | 5.91E-05 | 0.145508 |
| JVASP-36163 | CrInNi2 | 5.91E-05 | 0.090498 |
| JVASP-37271 | Sm2MgIr | 5.91E-05 | 0.245596 |
| JVASP-39804 | Ti2TcOs | 5.91E-05 | 0.069578 |
| JVASP-40403 | Be2RuAu | 5.91E-05 | 0.260356 |
| JVASP-40429 | K2SbAu | 5.91E-05 | 0.124436 |
| JVASP-54483 | Ta9Fe2S6 | 5.91E-05 | 0.067878 |
| JVASP-1017 | Tb | 5.92E-05 | 1.54238 |
| JVASP-15910 | LiMgPdSb | 5.92E-05 | 0.12029 |
| JVASP-16446 | LiAu3 | 5.92E-05 | 0.856563 |
| JVASP-16883 | Zr6Sb2Pt | 5.92E-05 | 0.034222 |
| JVASP-18364 | InBi | 5.92E-05 | 0.150862 |
| JVASP-21950 | HfB4Ir3 | 5.92E-05 | 0.178709 |
| JVASP-22600 | RbS | 5.92E-05 | 0.042534 |
| JVASP-29647 | Bi2Pb2Se5 | 5.92E-05 | 0.132529 |
| JVASP-40541 | LiYAu2 | 5.92E-05 | 0.207494 |
| JVASP-5779 | NBrHf | 5.92E-05 | 1.149819 |
| JVASP-8066 | ZnSnSb2 | 5.92E-05 | 0.019531 |
| JVASP-15653 | CaCuSb | 5.93E-05 | 0.055167 |
| JVASP-18315 | LiBi | 5.93E-05 | 0.419108 |
| JVASP-19811 | TaS2 | 5.93E-05 | 0.041662 |
| JVASP-29287 | Bi2SnTe4 | 5.93E-05 | 0.057182 |
| JVASP-41018 | Nd2IrPd | 5.93E-05 | 1.390454 |
| JVASP-8538 | CeNiSb2 | 5.93E-05 | 0.067204 |
| JVASP-1107 | Ag2Te | 5.94E-05 | 0.065646 |
| JVASP-12369 | Ba2Pb | 5.94E-05 | 0.029209 |
| JVASP-15040 | LiPd | 5.94E-05 | 0.360108 |
| JVASP-17845 | Pt3Pb | 5.94E-05 | 0.341473 |
| JVASP-18735 | Li2MgPb | 5.94E-05 | 0.165834 |
| JVASP-18778 | SmPt3 | 5.94E-05 | 1.116565 |
| JVASP-18813 | BaBiAu | 5.94E-05 | 0.200494 |
| JVASP-38015 | AgPb3 | 5.94E-05 | 0.2621 |
| JVASP-40059 | Li2NdIn | 5.94E-05 | 0.176185 |
| JVASP-40337 | Tb2IrPd | 5.94E-05 | 1.536297 |
| JVASP-41242 | HfScOs2 | 5.94E-05 | 0.112921 |
| JVASP-41734 | TaTlO3 | 5.94E-05 | 0.05451 |
| JVASP-51306 | GaAs2W | 5.94E-05 | 0.105872 |
| JVASP-5329 | O2F2Xe | 5.94E-05 | 0.004985 |
| JVASP-56312 | TlPd3 | 5.94E-05 | 0.0995 |
| JVASP-56621 | Tb3AlC | 5.94E-05 | 1.143762 |
| JVASP-1020 | Tc | 5.95E-05 | 1.26067 |
| JVASP-1124 | Cu2Se | 5.95E-05 | 0.08176 |
| JVASP-18487 | Te3Bi4 | 5.95E-05 | 0.023474 |
| JVASP-20921 | S3Fe2Rb | 5.95E-05 | 0.038153 |
| JVASP-2268 | ThTeO | 5.95E-05 | 0.094752 |
| JVASP-22715 | KCaBi | 5.95E-05 | 1.022735 |
| JVASP-35066 | CaSnHg | 5.95E-05 | 0.039301 |
| JVASP-35711 | GaSb | 5.95E-05 | 0.143418 |
| JVASP-37024 | TlCdSe2 | 5.95E-05 | 1.029039 |
| JVASP-38303 | RbNa2Sb | 5.95E-05 | 0.195243 |
| JVASP-41281 | NaCa2Tl | 5.95E-05 | 0.175614 |
| JVASP-51138 | Na2LiTa | 5.95E-05 | 0.062995 |
| JVASP-54573 | Ti3Ag | 5.95E-05 | 0.027073 |
| JVASP-15156 | DyBRh3 | 5.96E-05 | 0.920066 |
| JVASP-15799 | Li3Tl | 5.96E-05 | 0.38704 |
| JVASP-17695 | Na2CdPb | 5.96E-05 | 0.422718 |
| JVASP-17706 | Er3PbC | 5.96E-05 | 0.615969 |
| JVASP-17890 | Tb3PbC | 5.96E-05 | 0.518689 |
| JVASP-18814 | SrBiAu | 5.96E-05 | 0.182825 |
| JVASP-35007 | NbGeSb | 5.96E-05 | 0.034164 |
| JVASP-35491 | In2AgSe4 | 5.96E-05 | 0.03175 |
| JVASP-36263 | MgAgSb | 5.96E-05 | 0.131239 |
| JVASP-37693 | Ge2O3 | 5.96E-05 | 0.002753 |
| JVASP-38806 | NbTlO3 | 5.96E-05 | 0.017255 |
| JVASP-39840 | TiVRe2 | 5.96E-05 | 0.157699 |
| JVASP-40288 | SrCdHg2 | 5.96E-05 | 0.523705 |
| JVASP-40348 | TaAlFe2 | 5.96E-05 | 0.088171 |
| JVASP-41162 | HfAlRu2 | 5.96E-05 | 0.154458 |
| JVASP-56327 | YInAg2 | 5.96E-05 | 0.155278 |
| JVASP-8 | MoTe2 | 5.96E-05 | 0.025013 |
| JVASP-1123 | Cu2Se | 5.97E-05 | 0.073433 |
| JVASP-16767 | AlCuPt2 | 5.97E-05 | 0.068488 |
| JVASP-20161 | Al3Pt5 | 5.97E-05 | 0.158771 |
| JVASP-35013 | NdTlCd | 5.97E-05 | 0.10557 |
| JVASP-36459 | Ca3BiN | 5.97E-05 | 0.903765 |
| JVASP-36762 | AgBiO2 | 5.97E-05 | 0.021759 |
| JVASP-41274 | MgInIr2 | 5.97E-05 | 0.32448 |
| JVASP-41443 | Lu2ZnOs | 5.97E-05 | 1.617207 |
| JVASP-51314 | Re2AuSe | 5.97E-05 | 0.118684 |
| JVASP-72 | WS2 | 5.97E-05 | 0.01218 |
| JVASP-8455 | CaHgPb | 5.97E-05 | 0.107941 |
| JVASP-12101 | GeZrTe | 5.98E-05 | 0.014808 |
| JVASP-16408 | LiF6Ir | 5.98E-05 | 0.062233 |
| JVASP-18234 | Dy3TlC | 5.98E-05 | 0.562169 |
| JVASP-23424 | TaSiRh | 5.98E-05 | 0.029873 |
| JVASP-26961 | Sc5Pb3 | 5.98E-05 | 0.055089 |
| JVASP-35533 | TlPPt5 | 5.98E-05 | 0.086391 |
| JVASP-37222 | SmTh3 | 5.98E-05 | 1.302833 |
| JVASP-37738 | LiAl2Tc | 5.98E-05 | 0.271323 |
| JVASP-38829 | MgInRh2 | 5.98E-05 | 0.137124 |
| JVASP-39150 | Na2CdHg | 5.98E-05 | 0.457646 |
| JVASP-39850 | Cd2PdRh | 5.98E-05 | 0.048818 |
| JVASP-7998 | O3U | 5.98E-05 | 0.089115 |
| JVASP-16266 | Sm3TlC | 5.99E-05 | 0.308563 |
| JVASP-17506 | Nd3PbN | 5.99E-05 | 0.513814 |
| JVASP-18945 | PSm | 5.99E-05 | 2.249196 |
| JVASP-19593 | Ni2Y | 5.99E-05 | 0.652253 |
| JVASP-35194 | ZnGeO3 | 5.99E-05 | 0.009756 |
| JVASP-39311 | LuRhO3 | 5.99E-05 | 0.743438 |
| JVASP-51203 | ReRuBr | 5.99E-05 | 0.077335 |
| JVASP-16256 | Ca3TlN | 6.00E-05 | 0.193008 |
| JVASP-16880 | HfGaAu | 6.00E-05 | 0.043337 |
| JVASP-18560 | YSi2Rh3 | 6.00E-05 | 0.03678 |
| JVASP-35621 | Tl3VSe4 | 6.00E-05 | 0.04682 |
| JVASP-35698 | Ti2InCo | 6.00E-05 | 0.089263 |
| JVASP-37231 | SmAg2Hg | 6.00E-05 | 0.444314 |
| JVASP-38969 | TaGeRh | 6.00E-05 | 0.029198 |
| JVASP-39142 | LiSmAu2 | 6.00E-05 | 0.296537 |
| JVASP-39478 | Re3Ge | 6.00E-05 | 0.070866 |
| JVASP-40108 | CaThRh2 | 6.00E-05 | 0.089382 |
| JVASP-40573 | SmZnAu2 | 6.00E-05 | 0.103565 |
| JVASP-40907 | CaHg3 | 6.00E-05 | 0.074582 |
| JVASP-40977 | Ca2PdAu | 6.00E-05 | 0.094352 |
| JVASP-41417 | LiScTl2 | 6.00E-05 | 0.081012 |
| JVASP-41658 | LiNd2Ga | 6.00E-05 | 0.100045 |
| JVASP-41780 | Ba2AsAu | 6.00E-05 | 0.074384 |
| JVASP-51322 | AlTc2Pb | 6.00E-05 | 0.06815 |
| JVASP-54934 | Nd3AlC | 6.00E-05 | 1.111944 |
| JVASP-12309 | Sn7Ir5 | 6.01E-05 | 0.021361 |
| JVASP-15996 | PtO | 6.01E-05 | 0.184047 |
| JVASP-22016 | B7Y3W | 6.01E-05 | 0.022911 |
| JVASP-35027 | PdCl2 | 6.01E-05 | 0.076223 |
| JVASP-35074 | SmSe2 | 6.01E-05 | 0.061405 |
| JVASP-36497 | Sr3BiSb | 6.01E-05 | 0.110165 |
| JVASP-39092 | Si2OsRu | 6.01E-05 | 0.293395 |
| JVASP-40222 | LiDyTl2 | 6.01E-05 | 0.515474 |
| JVASP-56106 | As4C3 | 6.01E-05 | 0.040442 |
| JVASP-56771 | Li2AgSn | 6.01E-05 | 0.222271 |
| JVASP-937 | Nd | 6.01E-05 | 1.484398 |
| JVASP-16525 | GaPt3 | 6.02E-05 | 0.330848 |
| JVASP-19727 | SiRh | 6.02E-05 | 0.051864 |
| JVASP-23385 | NdZnSn | 6.02E-05 | 0.833388 |
| JVASP-35668 | TlN | 6.02E-05 | 0.044303 |
| JVASP-35808 | CaBe2As2 | 6.02E-05 | 0.020253 |
| JVASP-38333 | NdDy3 | 6.02E-05 | 1.374687 |
| JVASP-38616 | Hf3Au | 6.02E-05 | 0.066997 |
| JVASP-39864 | ThSnRu2 | 6.02E-05 | 0.10147 |
| JVASP-40525 | Sc2CuOs | 6.02E-05 | 0.079163 |
| JVASP-41563 | TiAlOs2 | 6.02E-05 | 0.105707 |
| JVASP-8448 | Nd3PbC | 6.02E-05 | 0.561486 |
| JVASP-890 | Ge | 6.02E-05 | 0.13469 |
| JVASP-1118 | CuBiSeO | 6.03E-05 | 0.10738 |
| JVASP-131 | SnS2 | 6.03E-05 | 0.011065 |
| JVASP-16055 | SrAsPt | 6.03E-05 | 0.067742 |
| JVASP-18119 | YAgPb | 6.03E-05 | 0.107464 |
| JVASP-19669 | SrRh2 | 6.03E-05 | 0.108039 |
| JVASP-41834 | Sc2ZnPt | 6.03E-05 | 0.318313 |
| JVASP-19668 | Nb3Sn | 6.04E-05 | 0.118289 |
| JVASP-36140 | TlN | 6.04E-05 | 0.033882 |
| JVASP-36674 | CdNi3N | 6.04E-05 | 0.045148 |
| JVASP-38637 | NiAu3 | 6.04E-05 | 0.048836 |
| JVASP-40436 | Er2NiOs | 6.04E-05 | 1.347636 |
| JVASP-41451 | NbAlOs2 | 6.04E-05 | 0.135363 |
| JVASP-1384 | LiPb | 6.05E-05 | 0.203531 |
| JVASP-16490 | W | 6.05E-05 | 0.11655 |
| JVASP-21889 | Sr3Hg2 | 6.05E-05 | 0.291843 |
| JVASP-25161 | Pb | 6.05E-05 | 0.160652 |
| JVASP-35214 | Na2ZnSe2 | 6.05E-05 | 0.020186 |
| JVASP-35984 | IrC | 6.05E-05 | 0.269159 |
| JVASP-36120 | HfPd3 | 6.05E-05 | 0.442231 |
| JVASP-40794 | TaAlPt | 6.05E-05 | 0.147392 |
| JVASP-41244 | Be2IrPd | 6.05E-05 | 0.445167 |
| JVASP-5098 | PtPb | 6.05E-05 | 0.163447 |
| JVASP-14601 | Sn | 6.06E-05 | 0.118492 |
| JVASP-17752 | NaPb3 | 6.06E-05 | 0.162581 |
| JVASP-18208 | Ag2InTb | 6.06E-05 | 0.082437 |
| JVASP-2062 | Se2TlBi | 6.06E-05 | 0.031248 |
| JVASP-22720 | Ba2SbAu | 6.06E-05 | 0.080827 |
| JVASP-28454 | Te2W | 6.06E-05 | 0.163564 |
| JVASP-36493 | Ba3BiP | 6.06E-05 | 0.988045 |
| JVASP-37603 | Sr3CdO4 | 6.06E-05 | 0.073511 |
| JVASP-38631 | NiAu3 | 6.06E-05 | 0.143199 |
| JVASP-38634 | Ni3Bi | 6.06E-05 | 0.197454 |
| JVASP-40603 | DyAgHg2 | 6.06E-05 | 1.407061 |
| JVASP-41159 | MgSnIr2 | 6.06E-05 | 0.129955 |
| JVASP-50293 | AgSnO3 | 6.06E-05 | 0.04926 |
| JVASP-14383 | InPd3 | 6.07E-05 | 0.117189 |
| JVASP-14812 | Sm | 6.07E-05 | 1.626735 |
| JVASP-17770 | PdPb2 | 6.07E-05 | 0.056559 |
| JVASP-18688 | CuRh2Sn | 6.07E-05 | 0.102565 |
| JVASP-18767 | Li2AlRh | 6.07E-05 | 0.316885 |
| JVASP-26362 | KNb4O5F | 6.07E-05 | 0.017949 |
| JVASP-30119 | MoO3 | 6.07E-05 | 0.001211 |
| JVASP-35707 | HfPt3 | 6.07E-05 | 0.473751 |
| JVASP-36460 | Ca3AsN | 6.07E-05 | 0.034323 |
| JVASP-36774 | HAuO2 | 6.07E-05 | 0.055991 |
| JVASP-39345 | PdAu3 | 6.07E-05 | 0.168589 |
| JVASP-40283 | SrLi2Pb | 6.07E-05 | 0.186403 |
| JVASP-51128 | ScHgW2 | 6.07E-05 | 0.103104 |
| JVASP-51217 | YRhW2 | 6.07E-05 | 0.123845 |
| JVASP-53970 | NdMg2Ag | 6.07E-05 | 0.628987 |
| JVASP-79710 | Na2HgBi | 6.07E-05 | 0.005553 |
| JVASP-8749 | GeRh3Sm2 | 6.07E-05 | 0.236577 |
| JVASP-27853 | Nb2CoS4 | 6.07E-05 | 0.15364 |
| JVASP-18340 | SrBi3 | 6.08E-05 | 0.224116 |
| JVASP-18740 | LiPt2 | 6.08E-05 | 0.241682 |
| JVASP-21678 | Be2W | 6.08E-05 | 0.043996 |
| JVASP-36286 | LiCdAs | 6.08E-05 | 0.949423 |
| JVASP-39356 | LiLa2Al | 6.08E-05 | 0.731362 |
| JVASP-39668 | Li2TlAg | 6.08E-05 | 0.190615 |
| JVASP-40060 | Ti2ZnRe | 6.08E-05 | 0.067214 |
| JVASP-40530 | LiYTl2 | 6.08E-05 | 0.088858 |
| JVASP-40592 | SmCdAu2 | 6.08E-05 | 0.876187 |
| JVASP-8122 | O2NaRu | 6.08E-05 | 0.06391 |
| JVASP-14622 | Ga | 6.09E-05 | 0.043979 |
| JVASP-16703 | DySbPd | 6.09E-05 | 0.052583 |
| JVASP-20561 | ScAs | 6.09E-05 | 0.050603 |
| JVASP-29975 | Bi2Te4Pb | 6.09E-05 | 0.053584 |
| JVASP-39218 | Li2MgGe | 6.09E-05 | 0.124437 |
| JVASP-39657 | Y2TlAg | 6.09E-05 | 0.103871 |
| JVASP-5365 | Cl3Cr | 6.09E-05 | 0.03008 |
| JVASP-5428 | GeTe4Bi2 | 6.09E-05 | 0.061669 |
| JVASP-16077 | Te4Pt3 | 6.10E-05 | 0.043628 |
| JVASP-16859 | Cu2ZnGeTe4 | 6.10E-05 | 0.017708 |
| JVASP-19846 | GaNi3 | 6.10E-05 | 0.091872 |
| JVASP-21663 | SmSbIr | 6.10E-05 | 0.117677 |
| JVASP-22148 | NdB4Os4 | 6.10E-05 | 0.056329 |
| JVASP-35057 | KMgAs | 6.10E-05 | 0.041227 |
| JVASP-38564 | Nd3Al | 6.10E-05 | 1.051609 |
| JVASP-41216 | Hf2CuOs | 6.10E-05 | 0.096411 |
| JVASP-17216 | LiGeRh2 | 6.11E-05 | 0.175544 |
| JVASP-18612 | Li2InAu | 6.11E-05 | 0.246 |
| JVASP-35893 | HfVSi | 6.11E-05 | 0.028832 |
| JVASP-36700 | BaWN3 | 6.11E-05 | 0.019694 |
| JVASP-4837 | O3Ga2 | 6.11E-05 | 0.004085 |
| JVASP-54 | MoS2 | 6.11E-05 | 0.010079 |
| JVASP-14822 | Tl | 6.12E-05 | 0.210804 |
| JVASP-20064 | SbLa | 6.12E-05 | 0.200506 |
| JVASP-29398 | F4V | 6.12E-05 | 0.045195 |
| JVASP-32749 | BiI | 6.12E-05 | 0.031504 |
| JVASP-36210 | YBiPd | 6.12E-05 | 0.140832 |
| JVASP-36880 | TlAgBr3 | 6.12E-05 | 0.012729 |
| JVASP-39536 | ZnCdS2 | 6.12E-05 | 0.006577 |
| JVASP-40457 | ThPbAu2 | 6.12E-05 | 0.109078 |
| JVASP-40498 | Sc2CuAu | 6.12E-05 | 0.076944 |
| JVASP-11005 | MgPd2 | 6.13E-05 | 0.071766 |
| JVASP-18042 | PtPb2 | 6.13E-05 | 0.063135 |
| JVASP-19601 | BTa2 | 6.13E-05 | 0.026111 |
| JVASP-32834 | InCl | 6.13E-05 | 0.002132 |
| JVASP-38848 | LiNd2Ru | 6.13E-05 | 0.865017 |
| JVASP-39794 | LiZrPt2 | 6.13E-05 | 0.102128 |
| JVASP-41271 | HfTc2Sn | 6.13E-05 | 0.078866 |
| JVASP-858 | Co | 6.13E-05 | 0.772058 |
| JVASP-14514 | Al2Sm | 6.14E-05 | 0.084537 |
| JVASP-17551 | ZrSiTe | 6.14E-05 | 0.042789 |
| JVASP-17733 | ScInPt2 | 6.14E-05 | 0.132079 |
| JVASP-18316 | DyBi | 6.14E-05 | 1.950925 |
| JVASP-22547 | BaGa2Te4 | 6.14E-05 | 0.007485 |
| JVASP-28376 | MoSeS | 6.14E-05 | 0.028482 |
| JVASP-28514 | MoSe2 | 6.14E-05 | 0.03227 |
| JVASP-29717 | Bi3Rh | 6.14E-05 | 0.010171 |
| JVASP-38569 | NdNi2Sb2 | 6.14E-05 | 0.044677 |
| JVASP-40047 | KTl2Bi | 6.14E-05 | 0.193291 |
| JVASP-40539 | LiAlPd2 | 6.14E-05 | 0.30319 |
| JVASP-41792 | Ca2SbAu | 6.14E-05 | 0.062446 |
| JVASP-7936 | NdCuSb2 | 6.14E-05 | 0.056517 |
| JVASP-6172 | Sc2CCl2 | 6.14E-05 | 0.710603 |
| JVASP-35899 | HfVGe | 6.15E-05 | 0.028261 |
| JVASP-35909 | KMgP | 6.15E-05 | 0.019624 |
| JVASP-36129 | HgPt3 | 6.15E-05 | 0.232739 |
| JVASP-36223 | TiGePt | 6.15E-05 | 0.137797 |
| JVASP-36754 | LiAuO2 | 6.15E-05 | 0.048743 |
| JVASP-36771 | AgBiO2 | 6.15E-05 | 0.024543 |
| JVASP-37797 | HfZnPt2 | 6.15E-05 | 0.068154 |
| JVASP-37875 | BPbO3 | 6.15E-05 | 0.015091 |
| JVASP-41554 | Y2NiIr | 6.15E-05 | 0.058097 |
| JVASP-54794 | SmAgPb | 6.15E-05 | 0.133089 |
| JVASP-13985 | AlAs | 6.16E-05 | 0.032242 |
| JVASP-25250 | N | 6.16E-05 | 0.015714 |
| JVASP-29634 | Te3Pt2 | 6.16E-05 | 0.033978 |
| JVASP-12059 | CuHO2 | 6.17E-05 | 0.066216 |
| JVASP-15523 | Zr2SbP | 6.17E-05 | 0.026496 |
| JVASP-210 | HfS2 | 6.17E-05 | 0.006236 |
| JVASP-216 | HfSe2 | 6.17E-05 | 0.017967 |
| JVASP-36297 | KAgO | 6.17E-05 | 0.135872 |
| JVASP-37415 | TaZnOs2 | 6.17E-05 | 0.123631 |
| JVASP-37907 | Ba2AgSb | 6.17E-05 | 0.064214 |
| JVASP-39974 | MgTaIr2 | 6.17E-05 | 0.136755 |
| JVASP-41062 | Hf2MoIr | 6.17E-05 | 0.198963 |
| JVASP-51336 | ReSnGe | 6.17E-05 | 0.130845 |
| JVASP-12018 | SrSbSe2F | 6.18E-05 | 0.061171 |
| JVASP-17598 | Pt3PbC | 6.18E-05 | 0.171365 |
| JVASP-35797 | PtC | 6.18E-05 | 0.153328 |
| JVASP-41008 | Nd2MgAl | 6.18E-05 | 0.770593 |
| JVASP-12069 | PZr2Te2 | 6.19E-05 | 0.046764 |
| JVASP-17452 | PZr2Te2 | 6.19E-05 | 0.046765 |
| JVASP-17559 | YCuSb2 | 6.19E-05 | 0.041304 |
| JVASP-18884 | Ag2Se | 6.19E-05 | 0.021451 |
| JVASP-19956 | SNd | 6.19E-05 | 3.087944 |
| JVASP-25666 | SmAsS | 6.19E-05 | 0.028216 |
| JVASP-39685 | Li2TlBi | 6.19E-05 | 0.386717 |
| JVASP-40004 | Be2PdRh | 6.19E-05 | 0.209283 |
| JVASP-20520 | Ni2Er | 6.20E-05 | 0.076776 |
| JVASP-20648 | NaPt2 | 6.20E-05 | 0.205319 |
| JVASP-25213 | Kr | 6.20E-05 | 0.150653 |
| JVASP-32741 | HfI3 | 6.20E-05 | 0.065898 |
| JVASP-35776 | BaC2 | 6.20E-05 | 0.00984 |
| JVASP-36761 | TlCuO2 | 6.20E-05 | 0.030743 |
| JVASP-37325 | TbYIr2 | 6.20E-05 | 0.143369 |
| JVASP-37918 | Ca3Tl | 6.20E-05 | 0.537713 |
| JVASP-39656 | TiNbRe2 | 6.20E-05 | 0.175725 |
| JVASP-41453 | NbAlFe2 | 6.20E-05 | 0.072511 |
| JVASP-51457 | V3Pt | 6.20E-05 | 0.137639 |
| JVASP-51471 | YPb2 | 6.20E-05 | 0.012746 |
| JVASP-54846 | ThGe2Os2 | 6.20E-05 | 0.040963 |
| JVASP-8063 | SbTe2Tl | 6.20E-05 | 0.030901 |
| JVASP-15997 | ReSi | 6.21E-05 | 0.101089 |
| JVASP-17376 | CdSbAu | 6.21E-05 | 0.198598 |
| JVASP-18860 | Li2InIr | 6.21E-05 | 0.272398 |
| JVASP-19862 | CaPt2 | 6.21E-05 | 0.134069 |
| JVASP-20422 | HfSb2 | 6.21E-05 | 0.070196 |
| JVASP-41217 | Mg2PdPt | 6.21E-05 | 0.288807 |
| JVASP-52132 | Tb2O3 | 6.21E-05 | 0.031797 |
| JVASP-7657 | V2Hf | 6.21E-05 | 0.577605 |
| JVASP-15572 | LiGa2Ir | 6.22E-05 | 0.207417 |
| JVASP-17414 | AlTiAu2 | 6.22E-05 | 0.148371 |
| JVASP-28387 | Te2W | 6.22E-05 | 0.101145 |
| JVASP-36720 | VCu3Te4 | 6.22E-05 | 0.072197 |
| JVASP-38290 | RbSbO3 | 6.22E-05 | 0.015123 |
| JVASP-39037 | SmTa3 | 6.22E-05 | 0.698191 |
| JVASP-7818 | P | 6.22E-05 | 0.05385 |
| JVASP-10703 | Cd3As2 | 6.23E-05 | 0.03057 |
| JVASP-14881 | NLa | 6.23E-05 | 0.06944 |
| JVASP-20427 | LuMg | 6.23E-05 | 1.739327 |
| JVASP-36721 | NbCu3Te4 | 6.23E-05 | 0.050922 |
| JVASP-54786 | NiHg4 | 6.23E-05 | 0.085987 |
| JVASP-5701 | CuAsSe2 | 6.23E-05 | 0.050488 |
| JVASP-7674 | ThTaN3 | 6.23E-05 | 0.961472 |
| JVASP-993 | Sb | 6.23E-05 | 0.079837 |
| JVASP-17815 | Pb | 6.24E-05 | 0.175542 |
| JVASP-1849 | Li2AgSb | 6.24E-05 | 0.485393 |
| JVASP-35769 | AlBi | 6.24E-05 | 0.316653 |
| JVASP-41125 | HfZnNi2 | 6.24E-05 | 0.115658 |
| JVASP-41845 | BeGaRh2 | 6.24E-05 | 0.267296 |
| JVASP-51291 | CaBiB | 6.24E-05 | 0.100778 |
| JVASP-1083 | Mg2Sn | 6.25E-05 | 0.114912 |
| JVASP-15242 | NdBRh3 | 6.25E-05 | 0.955292 |
| JVASP-15765 | SmRh3C | 6.25E-05 | 0.074581 |
| JVASP-18838 | InPd3 | 6.25E-05 | 0.116679 |
| JVASP-20219 | NiTa2 | 6.25E-05 | 0.026218 |
| JVASP-22685 | CdTe | 6.25E-05 | 0.087298 |
| JVASP-252 | PbO | 6.25E-05 | 0.01209 |
| JVASP-35742 | BaGaSnH | 6.25E-05 | 0.034528 |
| JVASP-36222 | TiO2 | 6.25E-05 | 0.008941 |
| JVASP-37209 | SiSnO3 | 6.25E-05 | 0.007584 |
| JVASP-38635 | KF2 | 6.25E-05 | 0.002943 |
| JVASP-13597 | W2N | 6.25E-05 | 0.078864 |
| JVASP-20371 | Nb3Sb | 6.26E-05 | 0.085898 |
| JVASP-36689 | SrWN3 | 6.26E-05 | 0.001363 |
| JVASP-38429 | K3Ir | 6.26E-05 | 0.536039 |
| JVASP-38647 | Ni3Sb | 6.26E-05 | 0.181224 |
| JVASP-40320 | SmAgHg2 | 6.26E-05 | 1.274046 |
| JVASP-40365 | CaMgTl2 | 6.26E-05 | 0.205372 |
| JVASP-51241 | BaMgOs2 | 6.26E-05 | 0.127895 |
| JVASP-52332 | LiBiS2 | 6.26E-05 | 0.362004 |
| JVASP-52617 | Sm2O3 | 6.26E-05 | 0.635188 |
| JVASP-77062 | Li2InSb | 6.26E-05 | 0.010806 |
| JVASP-81634 | Si2TcIr | 6.26E-05 | 0.007499 |
| JVASP-128 | PtSe2 | 6.27E-05 | 0.092828 |
| JVASP-14517 | Mo2Hf | 6.27E-05 | 0.110055 |
| JVASP-16321 | ScRh3C | 6.27E-05 | 0.033871 |
| JVASP-18629 | BaTl2 | 6.27E-05 | 0.098475 |
| JVASP-20251 | Ni2Er | 6.27E-05 | 0.076699 |
| JVASP-33718 | F | 6.27E-05 | 0.009121 |
| JVASP-35288 | ErInNi4 | 6.27E-05 | 0.107213 |
| JVASP-36185 | BiI3 | 6.27E-05 | 0.470922 |
| JVASP-38627 | NiAg3 | 6.27E-05 | 0.104202 |
| JVASP-39483 | Re3F | 6.27E-05 | 0.14952 |
| JVASP-41041 | Ta2OsW | 6.27E-05 | 0.151063 |
| JVASP-41122 | TbLiHg2 | 6.27E-05 | 0.260411 |
| JVASP-14612 | Zr | 6.28E-05 | 0.705141 |
| JVASP-15003 | Nb3Pt | 6.28E-05 | 0.081264 |
| JVASP-16762 | Al2W | 6.28E-05 | 0.062477 |
| JVASP-18133 | VFe2Ga | 6.28E-05 | 0.081529 |
| JVASP-20207 | Os2Th | 6.28E-05 | 0.157227 |
| JVASP-234 | NiO2 | 6.28E-05 | 0.083289 |
| JVASP-28576 | Te4W3Se2 | 6.28E-05 | 0.15119 |
| JVASP-29571 | Bi2Te5Pb2 | 6.28E-05 | 0.228205 |
| JVASP-37575 | YTlAg2 | 6.28E-05 | 0.102811 |
| JVASP-40313 | LiCa2In | 6.28E-05 | 0.097429 |
| JVASP-5668 | Cl4Ta | 6.28E-05 | 0.013707 |
| JVASP-25144 | P | 6.29E-05 | 0.053849 |
| JVASP-37962 | Ag3Rh | 6.29E-05 | 0.070812 |
| JVASP-41153 | Zr2CuOs | 6.29E-05 | 0.058933 |
| JVASP-51113 | SnIrSe2 | 6.29E-05 | 0.096981 |
| JVASP-56931 | Ti4CoBi2 | 6.29E-05 | 0.032313 |
| JVASP-17367 | HfCu2P2 | 6.30E-05 | 0.072204 |
| JVASP-18138 | NbSiAs | 6.30E-05 | 0.078562 |
| JVASP-22430 | NdB4Ir4 | 6.30E-05 | 0.098496 |
| JVASP-35883 | Hf2GaSb3 | 6.30E-05 | 0.062752 |
| JVASP-38642 | MgPb3 | 6.30E-05 | 0.143064 |
| JVASP-39920 | Dy2TlCd | 6.30E-05 | 0.086206 |
| JVASP-41765 | LiDy2Al | 6.30E-05 | 0.346863 |
| JVASP-4957 | TiPbO3 | 6.30E-05 | 0.004915 |
| JVASP-15164 | ZrGeTe | 6.31E-05 | 0.036047 |
| JVASP-17807 | Mg2Pb | 6.31E-05 | 0.36985 |
| JVASP-17933 | Er3TlC | 6.31E-05 | 0.604415 |
| JVASP-20488 | TbTl3 | 6.31E-05 | 2.003769 |
| JVASP-28541 | Mo3Se4S2 | 6.31E-05 | 0.008139 |
| JVASP-37304 | SrGaGeH | 6.31E-05 | 0.023868 |
| JVASP-39086 | Si3W | 6.31E-05 | 0.102973 |
| JVASP-54664 | ScIr3C | 6.31E-05 | 0.081519 |
| JVASP-76869 | Y2TlCu | 6.31E-05 | 0.009161 |
| JVASP-1056 | Zn | 6.32E-05 | 0.089864 |
| JVASP-22567 | O2Sn | 6.32E-05 | 0.001785 |
| JVASP-36656 | VPt | 6.32E-05 | 0.126808 |
| JVASP-41762 | CdGaRh2 | 6.32E-05 | 0.273899 |
| JVASP-19619 | CdPt | 6.33E-05 | 0.760434 |
| JVASP-19800 | V3Pd | 6.33E-05 | 0.046759 |
| JVASP-19845 | SeDy | 6.33E-05 | 2.27362 |
| JVASP-36811 | TlCuO2 | 6.33E-05 | 0.055818 |
| JVASP-5335 | NbSe2Cl2 | 6.33E-05 | 0.027018 |
| JVASP-76930 | Li2MgGa | 6.33E-05 | 0.008714 |
| JVASP-13550 | SiAs3 | 6.33E-05 | 0.045379 |
| JVASP-1002 | Si | 6.34E-05 | 0.066314 |
| JVASP-15130 | TbCo3B2 | 6.34E-05 | 0.613026 |
| JVASP-35806 | ScPt3C | 6.34E-05 | 0.106962 |
| JVASP-36040 | ErN | 6.34E-05 | 0.82803 |
| JVASP-39115 | ScAu3 | 6.34E-05 | 0.041081 |
| JVASP-39225 | SbPt3 | 6.34E-05 | 0.16994 |
| JVASP-27726 | PTe2Zr2 | 6.34E-05 | 0.020688 |
| JVASP-15884 | ScBIr3 | 6.35E-05 | 0.263338 |
| JVASP-36648 | MgTe | 6.35E-05 | 0.068085 |
| JVASP-41589 | V2CrOs | 6.35E-05 | 0.082394 |
| JVASP-5308 | MnO2 | 6.35E-05 | 0.089342 |
| JVASP-80997 | ZnNiPd2 | 6.35E-05 | 0.039095 |
| JVASP-19702 | MgHg | 6.36E-05 | 0.196919 |
| JVASP-39883 | BeAlIr2 | 6.36E-05 | 0.241522 |
| JVASP-40380 | Tc3Pt | 6.36E-05 | 0.085706 |
| JVASP-5851 | CrBrO | 6.36E-05 | 0.112904 |
| JVASP-16219 | Y3TlC | 6.37E-05 | 0.274721 |
| JVASP-20466 | Os2Th | 6.37E-05 | 0.15483 |
| JVASP-28452 | WS2 | 6.37E-05 | 0.039214 |
| JVASP-28507 | Te4Mo3S2 | 6.37E-05 | 0.097586 |
| JVASP-36235 | SrC2 | 6.37E-05 | 0.013354 |
| JVASP-37338 | SnPbO3 | 6.37E-05 | 0.005475 |
| JVASP-40061 | Ti3Pb | 6.37E-05 | 0.018514 |
| JVASP-51097 | TaFeSb | 6.37E-05 | 0.078306 |
| JVASP-16299 | Sm3AlN | 6.38E-05 | 0.712057 |
| JVASP-16640 | TePt | 6.38E-05 | 0.167652 |
| JVASP-19732 | TaN | 6.38E-05 | 0.106323 |
| JVASP-22652 | ScSbPd | 6.38E-05 | 0.020358 |
| JVASP-40253 | SmZn2Ag | 6.38E-05 | 1.442172 |
| JVASP-41757 | LiBiRh2 | 6.38E-05 | 0.35738 |
| JVASP-16591 | Ni3Sn | 6.39E-05 | 0.169705 |
| JVASP-36507 | Ba3BiSb | 6.39E-05 | 0.896102 |
| JVASP-40816 | NaLiPt | 6.39E-05 | 0.24305 |
| JVASP-51323 | B2IrCl | 6.39E-05 | 0.089592 |
| JVASP-57 | MoSe2 | 6.39E-05 | 0.016455 |
| JVASP-17541 | Dy3InC | 6.40E-05 | 1.041021 |
| JVASP-18817 | Ca3SbN | 6.40E-05 | 0.091875 |
| JVASP-19781 | SEr | 6.40E-05 | 2.141983 |
| JVASP-35079 | SrSnHg | 6.40E-05 | 0.042 |
| JVASP-5164 | CuI | 6.40E-05 | 0.053244 |
| JVASP-18149 | Nb3Pb | 6.41E-05 | 0.144564 |
| JVASP-28508 | Te2Mo2WSe2S2 | 6.41E-05 | 0.011343 |
| JVASP-4615 | SCuAg | 6.41E-05 | 0.895314 |
| JVASP-16929 | BiN | 6.42E-05 | 0.144813 |
| JVASP-35884 | Hf2TaN3 | 6.42E-05 | 0.055872 |
| JVASP-40519 | ScNbTc2 | 6.42E-05 | 0.185317 |
| JVASP-51558 | InCuPt2 | 6.42E-05 | 0.078429 |
| JVASP-51748 | NaH2Pd3 | 6.42E-05 | 0.168126 |
| JVASP-20562 | Nb3Sb | 6.43E-05 | 0.087223 |
| JVASP-38027 | SbBrO | 6.43E-05 | 0.050142 |
| JVASP-41822 | Er2IrPd | 6.43E-05 | 1.414211 |
| JVASP-41914 | Re3Pd | 6.43E-05 | 0.093938 |
| JVASP-51180 | CdPOs2 | 6.43E-05 | 0.165384 |
| JVASP-667 | C | 6.43E-05 | 0.005037 |
| JVASP-18913 | PtHg | 6.44E-05 | 0.04714 |
| JVASP-19645 | SrPd5 | 6.44E-05 | 0.212 |
| JVASP-19880 | NdIr2 | 6.44E-05 | 0.2701 |
| JVASP-20549 | AsLu | 6.44E-05 | 1.493027 |
| JVASP-36109 | BiB | 6.44E-05 | 0.159927 |
| JVASP-36138 | TlAs | 6.44E-05 | 0.146045 |
| JVASP-37612 | TbSmIr2 | 6.44E-05 | 1.749963 |
| JVASP-39202 | SbTeO3 | 6.44E-05 | 0.233705 |
| JVASP-40768 | FeSiW | 6.44E-05 | 0.03916 |
| JVASP-15998 | Sr3SnO | 6.45E-05 | 0.036483 |
| JVASP-16376 | AlPt | 6.45E-05 | 0.545091 |
| JVASP-18542 | Dy3AlC | 6.45E-05 | 1.117777 |
| JVASP-18606 | Li2GaAu | 6.45E-05 | 0.263322 |
| JVASP-19696 | HfW2 | 6.45E-05 | 0.957875 |
| JVASP-28493 | MoW2Se2S4 | 6.45E-05 | 0.016 |
| JVASP-36699 | CaMoN3 | 6.45E-05 | 0.010471 |
| JVASP-44 | FeSe | 6.45E-05 | 0.028826 |
| JVASP-56974 | Nb3Bi | 6.45E-05 | 0.03521 |
| JVASP-12001 | InBi | 6.46E-05 | 0.019192 |
| JVASP-14574 | SeEr | 6.46E-05 | 0.078097 |
| JVASP-17521 | Tb3TlC | 6.46E-05 | 0.718077 |
| JVASP-21 | SePb | 6.46E-05 | 0.235064 |
| JVASP-56561 | ScBi | 6.46E-05 | 0.605458 |
| JVASP-11985 | MgRe3 | 6.47E-05 | 0.030076 |
| JVASP-14958 | SSe | 6.47E-05 | 0.371845 |
| JVASP-18483 | P2Rh3 | 6.47E-05 | 0.038174 |
| JVASP-18935 | ZrW2 | 6.47E-05 | 0.130044 |
| JVASP-37625 | SrTlHg2 | 6.47E-05 | 0.123394 |
| JVASP-4438 | SiCrTe3 | 6.47E-05 | 0.037924 |
| JVASP-16438 | ScAg | 6.48E-05 | 0.020565 |
| JVASP-18005 | InSb | 6.48E-05 | 0.060477 |
| JVASP-18163 | In2Au | 6.48E-05 | 0.098417 |
| JVASP-20616 | TiSe2 | 6.48E-05 | 0.020248 |
| JVASP-36238 | MgTe | 6.48E-05 | 0.182823 |
| JVASP-36244 | PtN2 | 6.48E-05 | 0.235587 |
| JVASP-37520 | SrAlGeH | 6.48E-05 | 0.012153 |
| JVASP-53340 | Dy2O3 | 6.48E-05 | 0.099258 |
| JVASP-1125 | Cu2Se | 6.49E-05 | 0.072812 |
| JVASP-14993 | AsTb | 6.49E-05 | 0.265504 |
| JVASP-296 | SeSn | 6.49E-05 | 0.008418 |
| JVASP-5260 | Bi2TeI | 6.49E-05 | 0.008402 |
| JVASP-53299 | UNi2Sn | 6.49E-05 | 0.231523 |
| JVASP-816 | Al | 6.49E-05 | 0.159666 |
| JVASP-81775 | Hf2CuMo | 6.49E-05 | 0.006804 |
| JVASP-16206 | GeSb | 6.50E-05 | 0.020058 |
| JVASP-16442 | DyAuPb | 6.50E-05 | 0.138679 |
| JVASP-17362 | DyRh3C | 6.50E-05 | 0.841454 |
| JVASP-38116 | Rb2LiTlCl6 | 6.50E-05 | 0.021813 |
| JVASP-40522 | Ca2BiAu | 6.50E-05 | 0.086022 |
| JVASP-41848 | LiAlPt2 | 6.50E-05 | 0.156433 |
| JVASP-41932 | GaPt3 | 6.50E-05 | 0.034476 |
| JVASP-14738 | ScPt3 | 6.51E-05 | 0.182396 |
| JVASP-17543 | NiSb2 | 6.51E-05 | 0.05218 |
| JVASP-19861 | Rh2Sm | 6.51E-05 | 0.115191 |
| JVASP-20603 | DyTe | 6.51E-05 | 2.113339 |
| JVASP-32145 | IrC2 | 6.51E-05 | 0.156749 |
| JVASP-38870 | NdCdAu2 | 6.51E-05 | 0.211871 |
| JVASP-51208 | LiTaBe | 6.51E-05 | 0.08926 |
| JVASP-81240 | Li2MgGa | 6.51E-05 | 0.008809 |
| JVASP-14606 | Ag | 6.52E-05 | 0.196006 |
| JVASP-14987 | AsDy | 6.52E-05 | 2.288205 |
| JVASP-17409 | Zr2CuSb3 | 6.52E-05 | 0.046884 |
| JVASP-20084 | BW2 | 6.52E-05 | 0.066632 |
| JVASP-20502 | CeNi5 | 6.52E-05 | 0.053923 |
| JVASP-21774 | SrZnSb2 | 6.52E-05 | 0.063441 |
| JVASP-39446 | RuRh3 | 6.52E-05 | 0.260716 |
| JVASP-40842 | BaNaSb | 6.52E-05 | 0.343509 |
| JVASP-8079 | SbLu | 6.52E-05 | 1.03207 |
| JVASP-14507 | Mg2Sn | 6.53E-05 | 0.115447 |
| JVASP-15206 | Si2YRh2 | 6.53E-05 | 0.032456 |
| JVASP-17920 | Ge2YRu2 | 6.53E-05 | 0.030243 |
| JVASP-40811 | LiZnP | 6.53E-05 | 0.020731 |
| JVASP-581 | NbSe2 | 6.53E-05 | 0.013748 |
| JVASP-5863 | OBr4W | 6.53E-05 | 0.008088 |
| JVASP-1050 | Y | 6.54E-05 | 0.699863 |
| JVASP-15506 | TaSiAs | 6.54E-05 | 0.04243 |
| JVASP-15858 | Tb3AlC | 6.54E-05 | 0.027129 |
| JVASP-17886 | TePb | 6.54E-05 | 0.141471 |
| JVASP-40858 | Ba2BiAu | 6.54E-05 | 0.134203 |
| JVASP-56776 | BiTe | 6.54E-05 | 0.515965 |
| JVASP-1009 | Sn | 6.55E-05 | 0.08295 |
| JVASP-17195 | NPt | 6.55E-05 | 0.286528 |
| JVASP-17542 | PbTh | 6.55E-05 | 0.027015 |
| JVASP-23440 | Sr2AsAu | 6.55E-05 | 0.125857 |
| JVASP-16377 | AlPt | 6.56E-05 | 0.100131 |
| JVASP-260 | SPb | 6.56E-05 | 0.009993 |
| JVASP-35642 | KWO3 | 6.56E-05 | 0.010779 |
| JVASP-36659 | ZnCdSe2 | 6.56E-05 | 0.041251 |
| JVASP-40797 | TiSnPd | 6.56E-05 | 0.034499 |
| JVASP-82 | ZrS2 | 6.56E-05 | 0.003834 |
| JVASP-15837 | Hf2S | 6.57E-05 | 0.119276 |
| JVASP-36352 | DyBiPd | 6.57E-05 | 1.316535 |
| JVASP-40205 | CaSnS3 | 6.57E-05 | 0.020403 |
| JVASP-20370 | ScAs | 6.58E-05 | 0.046413 |
| JVASP-14991 | SeY | 6.59E-05 | 0.067676 |
| JVASP-36237 | RuO2 | 6.59E-05 | 0.103075 |
| JVASP-40111 | ErSnRu2 | 6.59E-05 | 0.489516 |
| JVASP-19746 | ZrTe | 6.60E-05 | 0.03685 |
| JVASP-28513 | MoW2Se6 | 6.60E-05 | 0.009911 |
| JVASP-29502 | PbS | 6.60E-05 | 0.001973 |
| JVASP-7636 | NiHg | 6.60E-05 | 0.028231 |
| JVASP-15855 | Er3AlC | 6.61E-05 | 0.885186 |
| JVASP-40095 | CaYHg2 | 6.61E-05 | 0.136 |
| JVASP-7717 | TiPt | 6.61E-05 | 1.396991 |
| JVASP-16797 | TiAu4 | 6.62E-05 | 0.074387 |
| JVASP-17278 | HfSiSe | 6.62E-05 | 0.047593 |
| JVASP-17767 | La3InB | 6.62E-05 | 0.963331 |
| JVASP-35891 | HfSb2 | 6.62E-05 | 0.062679 |
| JVASP-36197 | B2AsP | 6.62E-05 | 0.017697 |
| JVASP-368 | ZrS3 | 6.62E-05 | 0.004492 |
| JVASP-37911 | Ba2TlZn | 6.62E-05 | 0.108128 |
| JVASP-35727 | Ga2BiAs | 6.63E-05 | 0.1087 |
| JVASP-41211 | Mg2PdRh | 6.63E-05 | 0.09661 |
| JVASP-16452 | YAu | 6.64E-05 | 1.427055 |
| JVASP-20477 | Cu2Te | 6.64E-05 | 0.135582 |
| JVASP-20617 | LuMg | 6.64E-05 | 1.736031 |
| JVASP-365 | ZrSe2 | 6.64E-05 | 0.01393 |
| JVASP-51 | S2Mo | 6.64E-05 | 0.061647 |
| JVASP-51281 | NiBTe | 6.64E-05 | 0.078661 |
| JVASP-1116 | PbSe | 6.65E-05 | 0.194807 |
| JVASP-36023 | TcB | 6.65E-05 | 0.104888 |
| JVASP-40278 | Sr2SnHg | 6.65E-05 | 0.074205 |
| JVASP-18373 | HBr | 6.66E-05 | 0.090606 |
| JVASP-36348 | PdC | 6.66E-05 | 0.065124 |
| JVASP-56093 | LiInSn | 6.66E-05 | 0.062439 |
| JVASP-16307 | CuCdSb | 6.67E-05 | 0.251907 |
| JVASP-290 | SnS2 | 6.67E-05 | 0.009078 |
| JVASP-51126 | HgOsPb2 | 6.67E-05 | 0.248478 |
| JVASP-51267 | SiBPb | 6.67E-05 | 0.105277 |
| JVASP-40831 | HfAsRh | 6.68E-05 | 0.30977 |
| JVASP-13822 | OCl4W | 6.69E-05 | 0.003115 |
| JVASP-15952 | Er3GaC | 6.69E-05 | 0.914499 |
| JVASP-28543 | Mo2WS6 | 6.69E-05 | 0.020125 |
| JVASP-19954 | AsSm | 6.70E-05 | 0.348048 |
| JVASP-19985 | TeHg | 6.70E-05 | 0.100973 |
| JVASP-22729 | Sr2SbAu | 6.70E-05 | 0.050405 |
| JVASP-40766 | ZrFeSe | 6.70E-05 | 0.023483 |
| JVASP-40644 | SmInRh2 | 6.71E-05 | 0.173958 |
| JVASP-15921 | SbLuPt | 6.72E-05 | 0.087448 |
| JVASP-36137 | HW | 6.73E-05 | 0.136996 |
| JVASP-40818 | TiPtPb | 6.73E-05 | 0.128537 |
| JVASP-7736 | TaRu | 6.73E-05 | 0.296595 |
| JVASP-19734 | TeHg | 6.74E-05 | 0.109025 |
| JVASP-38342 | RbF3 | 6.74E-05 | 0.00023 |
| JVASP-18311 | SmBi | 6.75E-05 | 0.619972 |
| JVASP-40822 | ZrPtPb | 6.75E-05 | 0.11948 |
| JVASP-51280 | BiBCl | 6.75E-05 | 0.288457 |
| JVASP-15072 | PNd | 6.76E-05 | 2.031019 |
| JVASP-19960 | AsTh | 6.76E-05 | 0.42803 |
| JVASP-22735 | LiCaAs | 6.76E-05 | 0.017406 |
| JVASP-28405 | TeWS | 6.76E-05 | 0.011987 |
| JVASP-987 | Ru | 6.76E-05 | 0.33658 |
| JVASP-18604 | ErAuPb | 6.77E-05 | 0.171163 |
| JVASP-19849 | Cr2Hf | 6.77E-05 | 0.104842 |
| JVASP-25273 | Hg | 6.77E-05 | 0.069956 |
| JVASP-870 | Dy | 6.77E-05 | 1.616199 |
| JVASP-35623 | GaPt | 6.78E-05 | 0.115884 |
| JVASP-36402 | RuC | 6.78E-05 | 0.068763 |
| JVASP-28528 | Mo2WSe4S2 | 6.80E-05 | 0.006897 |
| JVASP-36243 | RbCuO | 6.80E-05 | 0.061296 |
| JVASP-36428 | ZrC | 6.80E-05 | 0.036243 |
| JVASP-15536 | SnLuAu | 6.81E-05 | 0.416386 |
| JVASP-40774 | TiSiPd | 6.81E-05 | 0.020391 |
| JVASP-35738 | CaZnSe2 | 6.82E-05 | 0.031821 |
| JVASP-40201 | CaSnS3 | 6.82E-05 | 0.003967 |
| JVASP-40826 | ZrSnPt | 6.82E-05 | 0.065072 |
| JVASP-4696 | SrLiP | 6.82E-05 | 0.008714 |
| JVASP-51293 | SrBiB | 6.82E-05 | 0.1648 |
| JVASP-14566 | SDy | 6.83E-05 | 2.118596 |
| JVASP-25084 | W | 6.83E-05 | 0.116482 |
| JVASP-38100 | Rb2NaTlCl6 | 6.83E-05 | 0.022917 |
| JVASP-39511 | SrMgSn | 6.83E-05 | 0.032712 |
| JVASP-40776 | ZrSiPd | 6.83E-05 | 0.031939 |
| JVASP-28500 | MoW2Se4S2 | 6.84E-05 | 0.008055 |
| JVASP-18703 | LiMg2Ga | 6.85E-05 | 0.086035 |
| JVASP-39308 | HgN | 6.86E-05 | 0.095147 |
| JVASP-51174 | SrHfRe2 | 6.86E-05 | 0.069905 |
| JVASP-837 | Bi | 6.86E-05 | 0.046755 |
| JVASP-919 | Mg | 6.86E-05 | 0.167703 |
| JVASP-10173 | SbTe | 6.87E-05 | 0.052623 |
| JVASP-14725 | U | 6.87E-05 | 0.219413 |
| JVASP-16125 | ThSiAu | 6.87E-05 | 0.324607 |
| JVASP-35541 | NbTl3Se4 | 6.87E-05 | 0.058079 |
| JVASP-39081 | SnPb | 6.87E-05 | 3.78612 |
| JVASP-15215 | MgAsAg | 6.88E-05 | 0.030322 |
| JVASP-28480 | Te2Mo2WSe4 | 6.88E-05 | 0.007647 |
| JVASP-37896 | CaAgHg2 | 6.88E-05 | 0.135805 |
| JVASP-38021 | AgBiS2 | 6.88E-05 | 0.13737 |
| JVASP-40824 | ZrSiPt | 6.88E-05 | 0.076618 |
| JVASP-18790 | Ta3Sn | 6.89E-05 | 0.089257 |
| JVASP-36653 | IrC | 6.89E-05 | 0.235931 |
| JVASP-40775 | TiFeSe | 6.89E-05 | 0.01919 |
| JVASP-12183 | VBr2 | 6.90E-05 | 0.008884 |
| JVASP-16604 | OPt | 6.90E-05 | 0.361664 |
| JVASP-40807 | TiNiGe | 6.90E-05 | 0.018444 |
| JVASP-12036 | CuSe | 6.91E-05 | 0.020039 |
| JVASP-40843 | SrCaSi | 6.91E-05 | 0.016544 |
| JVASP-1049 | Y | 6.92E-05 | 0.734518 |
| JVASP-18007 | PbSe | 6.92E-05 | 0.166813 |
| JVASP-29381 | Bi2O2Se | 6.92E-05 | 0.01511 |
| JVASP-51244 | ZrAlW | 6.92E-05 | 0.083087 |
| JVASP-1084 | LaTe | 6.95E-05 | 1.23537 |
| JVASP-37390 | SrGaSiH | 6.96E-05 | 0.022499 |
| JVASP-40798 | LiMgP | 6.96E-05 | 0.018911 |
| JVASP-7831 | LiGeIn | 6.96E-05 | 0.044245 |
| JVASP-41379 | LiScPd2 | 6.97E-05 | 0.168511 |
| JVASP-16593 | TlNi | 6.98E-05 | 0.065292 |
| JVASP-20614 | SbEr | 6.98E-05 | 1.281458 |
| JVASP-41149 | NdAgHg2 | 6.98E-05 | 1.333468 |
| JVASP-16304 | B2Au | 6.99E-05 | 0.301767 |
| JVASP-36287 | LuSb | 6.99E-05 | 1.992789 |
| JVASP-40788 | TiFeTe | 6.99E-05 | 0.023107 |
| JVASP-40834 | TiBiRh | 7.00E-05 | 0.08075 |
| JVASP-16605 | ZrOs | 7.01E-05 | 0.05852 |
| JVASP-4708 | B2Au | 7.01E-05 | 0.301763 |
| JVASP-19782 | SnSb | 7.02E-05 | 0.070291 |
| JVASP-36373 | CaC2 | 7.02E-05 | 0.014324 |
| JVASP-37009 | ThC | 7.02E-05 | 0.258683 |
| JVASP-40117 | AgBr | 7.02E-05 | 0.032758 |
| JVASP-40795 | NbAlPt | 7.02E-05 | 0.069965 |
| JVASP-36254 | NaAgO | 7.03E-05 | 0.030809 |
| JVASP-54581 | YPbAu | 7.03E-05 | 0.070057 |
| JVASP-36398 | AgN | 7.04E-05 | 0.060621 |
| JVASP-40743 | HfNiSn | 7.05E-05 | 0.063281 |
| JVASP-28410 | WSe2 | 7.06E-05 | 0.006357 |
| JVASP-35772 | LaBiPt | 7.06E-05 | 0.809701 |
| JVASP-40796 | ZrGePt | 7.06E-05 | 0.07895 |
| JVASP-51220 | SrScBe | 7.06E-05 | 0.304832 |
| JVASP-39497 | YMgTl | 7.07E-05 | 0.061705 |
| JVASP-76891 | Y2InCu | 7.08E-05 | 0.074174 |
| JVASP-18353 | ErBi | 7.10E-05 | 1.999871 |
| JVASP-51313 | GaReAs | 7.10E-05 | 0.139689 |
| JVASP-19770 | SbNd | 7.11E-05 | 1.185577 |
| JVASP-23 | CdTe | 7.12E-05 | 0.061212 |
| JVASP-39926 | DyThTc2 | 7.12E-05 | 1.193292 |
| JVASP-15030 | IrO2 | 7.13E-05 | 0.150725 |
| JVASP-20572 | Sn2Ir | 7.13E-05 | 0.134836 |
| JVASP-19649 | SSm | 7.14E-05 | 1.844464 |
| JVASP-19726 | SnTe | 7.14E-05 | 0.097744 |
| JVASP-25168 | Bi | 7.15E-05 | 0.020193 |
| JVASP-39508 | KYSn | 7.15E-05 | 0.052604 |
| JVASP-36179 | CdTe | 7.17E-05 | 0.80729 |
| JVASP-40821 | NbInPt | 7.18E-05 | 0.081252 |
| JVASP-17535 | ThAs | 7.19E-05 | 0.128065 |
| JVASP-40764 | ZrFeTe | 7.19E-05 | 0.024439 |
| JVASP-21195 | Mo | 7.20E-05 | 0.372761 |
| JVASP-36193 | BeSe | 7.22E-05 | 0.018396 |
| JVASP-39510 | YTlCd | 7.22E-05 | 0.129173 |
| JVASP-40772 | YNiP | 7.22E-05 | 0.01935 |
| JVASP-825 | Au | 7.22E-05 | 0.350009 |
| JVASP-18639 | ThBi | 7.23E-05 | 0.481934 |
| JVASP-28465 | W3Se4S2 | 7.23E-05 | 0.102697 |
| JVASP-40786 | TiGePt | 7.23E-05 | 0.073095 |
| JVASP-51345 | Ta2H | 7.23E-05 | 0.128186 |
| JVASP-40836 | ZrBiRh | 7.25E-05 | 0.113277 |
| JVASP-20385 | Sn2Ir | 7.26E-05 | 0.134834 |
| JVASP-40825 | TaTlPt | 7.26E-05 | 0.090152 |
| JVASP-18338 | Bi | 7.27E-05 | 0.019555 |
| JVASP-35987 | InBi | 7.27E-05 | 0.520729 |
| JVASP-5176 | CuBr | 7.27E-05 | 0.019973 |
| JVASP-20282 | AsLu | 7.30E-05 | 1.657882 |
| JVASP-51214 | BeTcSe | 7.30E-05 | 0.041382 |
| JVASP-16450 | ScAu | 7.31E-05 | 0.291781 |
| JVASP-19810 | AsEr | 7.31E-05 | 0.094231 |
| JVASP-25180 | Ca | 7.31E-05 | 0.015112 |
| JVASP-40763 | KBaP | 7.31E-05 | 0.01283 |
| JVASP-819 | Ar | 7.31E-05 | 0.069121 |
| JVASP-207 | SGe | 7.32E-05 | 0.0047 |
| JVASP-36272 | DySb | 7.33E-05 | 2.882752 |
| JVASP-51110 | InReGe | 7.33E-05 | 0.110301 |
| JVASP-19882 | ScPt | 7.35E-05 | 0.154075 |
| JVASP-40817 | NbTlPt | 7.35E-05 | 0.101443 |
| JVASP-19871 | ErCd | 7.37E-05 | 1.343355 |
| JVASP-35763 | GaBi | 7.37E-05 | 0.484019 |
| JVASP-19971 | SnAu | 7.39E-05 | 0.201446 |
| JVASP-25217 | Bi | 7.39E-05 | 0.026728 |
| JVASP-14487 | C2Nd | 7.40E-05 | 0.26527 |
| JVASP-18863 | NiZrBi | 7.40E-05 | 0.062888 |
| JVASP-901 | Ir | 7.40E-05 | 0.163729 |
| JVASP-40793 | ScCoTe | 7.42E-05 | 0.035872 |
| JVASP-51158 | TlCuIr | 7.44E-05 | 0.155722 |
| JVASP-18299 | TiNiSn | 7.45E-05 | 0.035177 |
| JVASP-36413 | BaSiC | 7.45E-05 | 0.185877 |
| JVASP-14644 | Pd | 7.46E-05 | 0.114495 |
| JVASP-4349 | TeHg | 7.49E-05 | 0.108963 |
| JVASP-28409 | Te2Mo | 7.51E-05 | 0.002669 |
| JVASP-15966 | RhSbHf | 7.53E-05 | 0.050346 |
| JVASP-40829 | VInPt | 7.57E-05 | 0.033888 |
| JVASP-40770 | YCdGa | 7.59E-05 | 0.137185 |
| JVASP-1014 | Ta | 7.62E-05 | 0.107696 |
| JVASP-51127 | HfMnTl | 7.63E-05 | 0.069309 |
| JVASP-40800 | ZrSnPd | 7.64E-05 | 0.037223 |
| JVASP-14837 | V | 7.65E-05 | 0.304907 |
| JVASP-15923 | Ta2N | 7.68E-05 | 0.075778 |
| JVASP-40801 | VGaPt | 7.68E-05 | 0.076494 |
| JVASP-51106 | KHfNi | 7.73E-05 | 0.070482 |
| JVASP-54893 | HfCoSb | 7.75E-05 | 0.117444 |
| JVASP-1007 | Sn | 7.80E-05 | 0.111098 |
| JVASP-972 | Pt | 7.80E-05 | 0.21783 |
| JVASP-934 | Nb | 7.81E-05 | 0.032339 |
| JVASP-39496 | NaYSi | 7.84E-05 | 0.017159 |
| JVASP-14604 | Ba | 7.89E-05 | 0.244914 |
| JVASP-36385 | Ca2Si | 7.90E-05 | 0.026598 |
| JVASP-25234 | Bi | 7.91E-05 | 0.021831 |
| JVASP-961 | Pb | 7.92E-05 | 0.247955 |
| JVASP-25135 | Tb | 7.96E-05 | 2.686278 |
| JVASP-16288 | TlSe | 7.97E-05 | 0.167932 |
| JVASP-25114 | K | 7.97E-05 | 0.507147 |
| JVASP-984 | Rh | 7.97E-05 | 0.509377 |
| JVASP-867 | Cu | 7.99E-05 | 0.253143 |
| JVASP-943 | Ni | 8.00E-05 | 0.060491 |
| JVASP-25228 | Bi | 8.25E-05 | 0.233197 |
| JVASP-16087 | W | 8.27E-05 | 0.350901 |
| JVASP-963 | Pd | 8.40E-05 | 0.43309 |
| JVASP-36268 | Mn2Ge | 8.63E-05 | 0.054668 |
| JVASP-16082 | Nb | 8.79E-05 | 0.077258 |
| JVASP-25227 | Bi | 8.79E-05 | 0.664482 |
| JVASP-25346 | Nb | 8.79E-05 | 0.077258 |
| JVASP-15717 | Ta | 8.88E-05 | 0.093674 |
| JVASP-25320 | Ta | 8.88E-05 | 0.093674 |
| JVASP-16333 | Dy | 8.94E-05 | 2.327628 |
| JVASP-16335 | Er | 9.48E-05 | 2.5324 |
| JVASP-60241 | Ge3Sb2O9 | 0.000111 | 0.016012 |
| JVASP-21193 | Ne | 0.000117 | 0.039837 |
| JVASP-28027 | Bi4Te3S8 | 0.00012 | 0.039286 |
| JVASP-6823 | NbCl4 | 0.000663 | 0.013409 |
| JVASP-6136 | IrCl3 | 0.000945 | 0.063215 |
| JVASP-27956 | Mg3H2O4 | 0.001263 | 0.14456 |
| JVASP-20026 | BrF4Rb | 0.001267 | 0.001778 |
| JVASP-60505 | AlH3O3 | 0.001358 | 0.025092 |
| JVASP-31378 | SrSbSe2F | 0.001631 | 1.769866 |
| JVASP-28112 | CdInGaS4 | 0.001666 | 0.164156 |
| JVASP-14420 | NbSe2Br2 | 0.001676 | 0.010372 |
| JVASP-60507 | TeH2O4 | 0.002008 | 0.002116 |
| JVASP-60504 | HO2Sc | 0.002009 | 0.012084 |
| JVASP-27915 | Te4O9 | 0.002392 | 0.002492 |
| JVASP-6385 | InAgP2S6 | 0.002525 | 0.005335 |
| JVASP-6004 | BBr3 | 0.002831 | 0.002679 |
| JVASP-13616 | AsCl5 | 0.002863 | 0.002835 |
| JVASP-6502 | SiI3 | 0.002921 | 0.002921 |
| JVASP-6202 | IrBr3 | 0.003128 | 0.056488 |
| JVASP-6631 | CaN | 0.003244 | 0.032456 |
| JVASP-60249 | O2Te | 0.003348 | 0.003662 |
| JVASP-27780 | ZrTiSe4 | 0.003686 | 0.716111 |
| JVASP-6091 | PBr3 | 0.003748 | 0.003455 |
| JVASP-19550 | SiCl4 | 0.00381 | 0.00371 |
| JVASP-19552 | Br2O | 0.004134 | 0.003248 |
| JVASP-6334 | Ta2CS2 | 0.00414 | 0.022635 |
| JVASP-60549 | BiBr3 | 0.004223 | 0.004228 |
| JVASP-6256 | NbS2Cl2 | 0.004306 | 0.013247 |
| JVASP-5917 | PbS | 0.004468 | 0.008625 |
| JVASP-6517 | GaN | 0.004476 | 0.007063 |
| JVASP-6244 | TiPbO3 | 0.004486 | 0.011223 |
| JVASP-27957 | Mg2H2O3 | 0.004531 | 0.020458 |
| JVASP-19992 | PtTaTe5 | 0.00464 | 0.01356 |
| JVASP-6208 | SrHI | 0.004976 | 0.041554 |
| JVASP-28095 | Pb2O3 | 0.005883 | 0.007459 |
| JVASP-28262 | GaHO2 | 0.005924 | 0.119085 |
| JVASP-6418 | AlBr3 | 0.006051 | 0.006085 |
| JVASP-27889 | AgClO4 | 0.006071 | 0.010171 |
| JVASP-13623 | ZrGeTe | 0.006229 | 0.118139 |
| JVASP-60340 | BaAl2Cl8 | 0.006394 | 0.006689 |
| JVASP-6106 | BI3 | 0.006575 | 0.006575 |
| JVASP-6514 | NbI5 | 0.006599 | 0.006923 |
| JVASP-6448 | S5N6 | 0.006612 | 0.006908 |
| JVASP-6403 | AsBr3 | 0.006837 | 0.007083 |
| JVASP-6532 | SbCl5 | 0.007139 | 0.006939 |
| JVASP-19584 | Tl2SnAs2S6 | 0.0078 | 0.007751 |
| JVASP-6049 | TcS2 | 0.008138 | 0.010553 |
| JVASP-60300 | NdBr3 | 0.008415 | 0.006248 |
| JVASP-28153 | HgF | 0.008454 | 0.039184 |
| JVASP-31377 | Br2NbO | 0.008528 | 0.013914 |
| JVASP-6223 | OsCl2O | 0.008594 | 0.044138 |
| JVASP-20005 | SbCl5 | 0.00888 | 0.002932 |
| JVASP-60510 | SrAl2Cl8 | 0.009193 | 0.009193 |
| JVASP-6307 | MgPSe3 | 0.009236 | 0.016455 |
| JVASP-6238 | TaTe4Ir | 0.009315 | 0.024664 |
| JVASP-14432 | AlHO2 | 0.009812 | 0.045458 |
| JVASP-6127 | RuBr3 | 0.009821 | 0.047243 |
| JVASP-6478 | S3N2Cl2 | 0.009953 | 0.009054 |
| JVASP-7024 | Na4LiN2 | 0.010088 | 0.011565 |
| JVASP-5929 | SnSe | 0.010946 | 0.010421 |
| JVASP-5905 | BCl3 | 0.011799 | 0.011052 |
| JVASP-28080 | Bi2Pb2Se5 | 0.012031 | 0.0118 |
| JVASP-6085 | RhBr3 | 0.012198 | 0.051329 |
| JVASP-6799 | Ta3TeI7 | 0.012386 | 0.011568 |
| JVASP-28152 | GeSe | 0.012478 | 0.009686 |
| JVASP-6115 | AlI3 | 0.012824 | 0.012624 |
| JVASP-27982 | ZnC2S2O6F6 | 0.012827 | 0.012227 |
| JVASP-6142 | ScCl3 | 0.01291 | 0.01291 |
| JVASP-6025 | SnSe | 0.013035 | 0.014471 |
| JVASP-13636 | Nb2CS2 | 0.013047 | 0.026629 |
| JVASP-28223 | FeC2O6 | 0.013103 | 0.112715 |
| JVASP-6796 | Ta3SeI7 | 0.013153 | 0.012152 |
| JVASP-6526 | Nb2CS2 | 0.013247 | 0.025319 |
| JVASP-31353 | InBi | 0.013261 | 0.718113 |
| JVASP-27958 | MgH2O2 | 0.014097 | 0.013444 |
| JVASP-6382 | Nb3TeCl7 | 0.01411 | 0.02098 |
| JVASP-6808 | SiH | 0.014145 | 0.036342 |
| JVASP-31375 | PdSeO3 | 0.014327 | 0.075567 |
| JVASP-60337 | Ag3SnP7 | 0.014698 | 0.013798 |
| JVASP-6007 | PbO | 0.014728 | 0.013582 |
| JVASP-60236 | Nb2O5 | 0.015034 | 0.011802 |
| JVASP-6094 | BiI3 | 0.015362 | 0.017378 |
| JVASP-27755 | F4Pb | 0.015555 | 0.018321 |
| JVASP-6028 | SnSe | 0.015938 | 0.014639 |
| JVASP-27885 | GeS | 0.016023 | 0.016145 |
| JVASP-60604 | SeSn | 0.016571 | 0.018593 |
| JVASP-13611 | PI2 | 0.01747 | 0.016823 |
| JVASP-28268 | GeTe | 0.018305 | 0.019197 |
| JVASP-60776 | InS | 0.018308 | 0.034334 |
| JVASP-6274 | Bi2Te2S | 0.019528 | 0.027201 |
| JVASP-13624 | Ni2SbTe2 | 0.019825 | 0.023602 |
| JVASP-13600 | ZrFeCl6 | 0.020678 | 0.040657 |
| JVASP-5968 | SbI3 | 0.020818 | 0.026198 |
| JVASP-60607 | Al2Te5 | 0.021118 | 0.016571 |
| JVASP-6364 | AsCl3 | 0.02125 | 0.021454 |
| JVASP-60558 | Br3Y | 0.021292 | 0.030442 |
| JVASP-6298 | Bi2Te2Se | 0.02163 | 0.029955 |
| JVASP-6394 | TaI2Cl2 | 0.021957 | 0.021657 |
| JVASP-6160 | TaI2O | 0.022554 | 0.02286 |
| JVASP-6076 | TlF | 0.022561 | 0.022407 |
| JVASP-6349 | ZrCl | 0.023421 | 0.508522 |
| JVASP-19517 | NaMnP | 0.023453 | 0.064627 |
| JVASP-6139 | PCl3 | 0.023917 | 0.023617 |
| JVASP-5874 | PtO2 | 0.024175 | 0.081018 |
| JVASP-6484 | SNCl | 0.024346 | 0.024246 |
| JVASP-6508 | PdSCl | 0.024493 | 0.042204 |
| JVASP-60529 | Br6Li2U | 0.024658 | 0.130957 |
| JVASP-6766 | HfFeCl6 | 0.02535 | 0.040716 |
| JVASP-19549 | LiBiO2 | 0.025925 | 0.021982 |
| JVASP-6361 | SbSeI | 0.02605 | 0.026487 |
| JVASP-19540 | CClN | 0.026496 | 0.027779 |
| JVASP-6118 | TiI3 | 0.027337 | 0.044537 |
| JVASP-6220 | BiClO | 0.027772 | 0.035927 |
| JVASP-6262 | GaTeCl | 0.028067 | 0.030074 |
| JVASP-13588 | ScHCl | 0.028099 | 0.030656 |
| JVASP-19989 | SnS | 0.028785 | 0.010183 |
| JVASP-31367 | ZrBr | 0.0298 | 0.321447 |
| JVASP-6943 | CrCl3 | 0.029988 | 0.025105 |
| JVASP-8026 | Ni5U | 0.030509 | 0.205783 |
| JVASP-20012 | Br2CoO2Sr2 | 0.030962 | 0.069086 |
| JVASP-60543 | AgGaP2Se6 | 0.032155 | 0.033802 |
| JVASP-6445 | SN | 0.032451 | 0.039658 |
| JVASP-60573 | F2O2W | 0.032466 | 0.001373 |
| JVASP-6358 | TlPt2S3 | 0.033639 | 0.036586 |
| JVASP-5872 | TaS2 | 0.033948 | 0.032089 |
| JVASP-28013 | Tl2O | 0.034671 | 0.018042 |
| JVASP-27864 | Ti2Te2P | 0.035939 | 0.044306 |
| JVASP-5888 | SnO | 0.036031 | 0.036946 |
| JVASP-5938 | Te2Rh | 0.03864 | 0.077958 |
| JVASP-14224 | RbTe6 | 0.038684 | 0.065772 |
| JVASP-6229 | BiTeI | 0.039063 | 0.041127 |
| JVASP-6295 | HfCl4 | 0.039477 | 0.039377 |
| JVASP-27971 | CoCl2O8 | 0.039538 | 0.113206 |
| JVASP-60494 | ClRe3Se4 | 0.039832 | 0.044432 |
| JVASP-6082 | Te2I | 0.040037 | 0.052299 |
| JVASP-6472 | HS7N | 0.040892 | 0.044746 |
| JVASP-14427 | YI3 | 0.041032 | 0.039143 |
| JVASP-6826 | Bi2Se2Te | 0.041619 | 0.043971 |
| JVASP-27775 | Te2Au | 0.042953 | 0.03034 |
| JVASP-31420 | As2Te3 | 0.043007 | 0.048803 |
| JVASP-5956 | TaSe2 | 0.043828 | 0.045345 |
| JVASP-6163 | BiTeCl | 0.044032 | 0.04102 |
| JVASP-27724 | Sb2Te3 | 0.044375 | 0.049755 |
| JVASP-6376 | TlTe3Pt2 | 0.045321 | 0.038421 |
| JVASP-27836 | Se | 0.045535 | 0.045514 |
| JVASP-60536 | Cl3Mo | 0.045789 | 0.054866 |
| JVASP-6058 | Te2Br | 0.045988 | 0.04806 |
| JVASP-60484 | Br2Hg | 0.047115 | 0.046697 |
| JVASP-14456 | CoO2 | 0.04805 | 0.05664 |
| JVASP-60595 | GaGeTe | 0.048158 | 0.048894 |
| JVASP-27778 | Bi2Pt | 0.048629 | 0.078622 |
| JVASP-6148 | RhCl3 | 0.049195 | 0.088995 |
| JVASP-20029 | AuCN | 0.049326 | 0.219689 |
| JVASP-31373 | Bi2Se3 | 0.049722 | 0.053522 |
| JVASP-13602 | PtI2 | 0.051218 | 0.054218 |
| JVASP-60530 | GeI2Y2 | 0.053983 | 0.06573 |
| JVASP-20035 | Rb3Mo2Cl9 | 0.054281 | 0.075281 |
| JVASP-5995 | PbI2 | 0.054452 | 0.056856 |
| JVASP-19557 | PtS2Cl6 | 0.056649 | 0.059125 |
| JVASP-20040 | F2Kr | 0.057017 | 0.057583 |
| JVASP-6088 | CrBr3 | 0.057367 | 0.031567 |
| JVASP-19999 | I2Pb | 0.058 | 0.060504 |
| JVASP-28252 | Mg2SiO4 | 0.058405 | 0.087118 |
| JVASP-14417 | AgI | 0.058543 | 0.09804 |
| JVASP-19536 | AuSeBr | 0.059097 | 0.05827 |
| JVASP-5887 | CdBr2 | 0.060095 | 0.078315 |
| JVASP-6775 | P3Se4I2 | 0.063619 | 0.063619 |
| JVASP-28199 | VC2O6 | 0.063969 | 0.071634 |
| JVASP-6109 | MoBr3 | 0.064281 | 0.07386 |
| JVASP-6154 | ClF | 0.064817 | 0.068374 |
| JVASP-60526 | I2Pb | 0.065503 | 0.067733 |
| JVASP-60491 | I2Pb | 0.065687 | 0.068657 |
| JVASP-60493 | I2Pb | 0.065798 | 0.068069 |
| JVASP-60293 | PdI2 | 0.065974 | 0.063851 |
| JVASP-60443 | Cl3Cr | 0.066414 | 0.040314 |
| JVASP-5947 | NbS2 | 0.066696 | 0.066402 |
| JVASP-27773 | Cl2Zn | 0.067647 | 0.094194 |
| JVASP-19510 | NbSe2 | 0.068275 | 0.071888 |
| JVASP-5965 | MgI2 | 0.069074 | 0.072733 |
| JVASP-6430 | HgCl2 | 0.069212 | 0.069212 |
| JVASP-6217 | BiBrO | 0.070978 | 0.084466 |
| JVASP-60291 | ThI4 | 0.072527 | 0.079906 |
| JVASP-60488 | ReS2 | 0.073422 | 0.076112 |
| JVASP-6193 | InClO | 0.073883 | 0.053259 |
| JVASP-13632 | TiCl3 | 0.074026 | 0.075268 |
| JVASP-76195 | CrI3 | 0.074062 | 0.085168 |
| JVASP-6022 | ReS2 | 0.074399 | 0.090046 |
| JVASP-60280 | Br4U | 0.075908 | 0.067218 |
| JVASP-27912 | FeO2 | 0.076271 | 0.14535 |
| JVASP-60477 | Cl3Ru | 0.076873 | 0.106191 |
| JVASP-6097 | VCl3 | 0.07837 | 0.07287 |
| JVASP-6829 | CrBrO | 0.082331 | 0.121993 |
| JVASP-27923 | TeAuI | 0.083794 | 0.082896 |
| JVASP-28295 | NiS2 | 0.086024 | 0.068198 |
| JVASP-27906 | CrClO | 0.089097 | 0.15139 |
| JVASP-5977 | NiTe2 | 0.093091 | 0.084165 |
| JVASP-6742 | FeCl3 | 0.093992 | 0.119591 |
| JVASP-60357 | BrOV | 0.095897 | 0.089597 |
| JVASP-6214 | BiIO | 0.096139 | 0.105441 |
| JVASP-14445 | BiOF | 0.098843 | 0.105965 |
| JVASP-28280 | AgBiO2 | 0.102432 | 0.152822 |
| JVASP-60261 | BrCu | 0.104372 | 0.109792 |
| JVASP-5914 | ReSe2 | 0.105112 | 0.109836 |
| JVASP-28236 | FeCoO4 | 0.105168 | 0.144441 |
| JVASP-6241 | KMnP | 0.105453 | 0.188765 |
| JVASP-60522 | Te5U | 0.105641 | 0.120938 |
| JVASP-19998 | CdCl2 | 0.108201 | 0.174057 |
| JVASP-28296 | SnO2 | 0.1107 | 0.11574 |
| JVASP-6397 | CrI2 | 0.110741 | 0.119044 |
| JVASP-6187 | AuI | 0.115668 | 0.103617 |
| JVASP-13589 | CaHI | 0.116025 | 0.115425 |
| JVASP-13596 | AuSe | 0.116453 | 0.106766 |
| JVASP-6052 | CaI2 | 0.118177 | 0.129872 |
| JVASP-60349 | Br7STa3 | 0.121752 | 0.125652 |
| JVASP-28136 | PtCl2 | 0.123397 | 0.128029 |
| JVASP-60578 | CrP2S7 | 0.124549 | 0.125549 |
| JVASP-5953 | Te2Ir | 0.125203 | 0.125203 |
| JVASP-19524 | UI3 | 0.128671 | 0.133849 |
| JVASP-13539 | Sb | 0.129139 | 0.12937 |
| JVASP-13590 | CaHBr | 0.130566 | 0.368697 |
| JVASP-19539 | AuClTe2 | 0.133376 | 0.133329 |
| JVASP-20002 | Bi | 0.137551 | 0.150274 |
| JVASP-20004 | HgI2 | 0.139157 | 0.125942 |
| JVASP-6079 | InSe | 0.139707 | 0.148009 |
| JVASP-60392 | CrPS4 | 0.139711 | 0.148557 |
| JVASP-6877 | GaSe | 0.141088 | 0.17224 |
| JVASP-17880 | Dy3GaC | 0.142287 | 0.824101 |
| JVASP-27756 | AuBr | 0.14578 | 0.145238 |
| JVASP-28065 | AgBiP2Se6 | 0.146936 | 0.161488 |
| JVASP-14460 | Cr3O8 | 0.15112 | 0.154834 |
| JVASP-60589 | NiTeO4 | 0.156273 | 0.170672 |
| JVASP-60483 | NbCoTe2 | 0.157499 | 0.165356 |
| JVASP-28212 | TiCo3O8 | 0.157982 | 0.204178 |
| JVASP-60582 | CoTeO4 | 0.161651 | 0.210712 |
| JVASP-6922 | MnO2 | 0.162062 | 0.166936 |
| JVASP-60257 | BrCu | 0.164457 | 0.173771 |
| JVASP-653 | WSe2 | 0.168069 | 0.166408 |
| JVASP-14458 | TeO3 | 0.175532 | 0.174432 |
| JVASP-6034 | CoBr2 | 0.176691 | 0.176691 |
| JVASP-13628 | FeS | 0.177331 | 0.216519 |
| JVASP-5911 | SiTe2 | 0.178503 | 0.188354 |
| JVASP-6145 | US3 | 0.187525 | 0.213839 |
| JVASP-60294 | INU | 0.18761 | 0.197033 |
| JVASP-16235 | B2NiMo2 | 0.188305 | 0.044605 |
| JVASP-5935 | NiO2 | 0.191179 | 0.219315 |
| JVASP-6031 | MnI2 | 0.191259 | 0.206025 |
| JVASP-20007 | Cl2Mg | 0.191293 | 0.190805 |
| JVASP-20017 | MnI2 | 0.191409 | 0.205755 |
| JVASP-27834 | Ga | 0.202293 | 0.200543 |
| JVASP-27913 | MnH2O2 | 0.207198 | 0.219177 |
| JVASP-652 | WSe2 | 0.209703 | 0.208703 |
| JVASP-31379 | CoO2 | 0.210452 | 0.144074 |
| JVASP-651 | WSe2 | 0.211665 | 0.211365 |
| JVASP-6889 | CrSe2 | 0.215774 | 0.508003 |
| JVASP-6544 | SiH4 | 0.21754 | 0.307041 |
| JVASP-6664 | CaMnSi | 0.217891 | 0.216609 |
| JVASP-60254 | CuInP2Se6 | 0.220738 | 0.421031 |
| JVASP-36229 | ThH2 | 0.221417 | 0.283171 |
| JVASP-14419 | CoI2 | 0.22708 | 0.247908 |
| JVASP-6055 | FeCl2 | 0.231264 | 0.211823 |
| JVASP-27899 | ZrP2H2O6 | 0.231283 | 0.230983 |
| JVASP-27865 | Y2C | 0.231792 | 0.262843 |
| JVASP-27920 | NiI2 | 0.23254 | 0.241264 |
| JVASP-60286 | AlI2Pd5 | 0.239406 | 0.247669 |
| JVASP-6232 | CdCl2 | 0.24118 | 0.295471 |
| JVASP-13576 | 2-Feb | 0.248131 | 0.229413 |
| JVASP-27766 | CrAgP2S6 | 0.254445 | 0.255745 |
| JVASP-6676 | RbMnP | 0.256968 | 0.164701 |
| JVASP-6652 | RbMnAs | 0.267491 | 0.150812 |
| JVASP-28291 | CoS2 | 0.271592 | 0.30779 |
| JVASP-6319 | CrSiTe3 | 0.272424 | 0.272324 |
| JVASP-6100 | AlCl3 | 0.276208 | 0.282051 |
| JVASP-60391 | CoH2SeO4 | 0.277854 | 0.244754 |
| JVASP-60244 | FeSe | 0.280131 | 0.292152 |
| JVASP-5944 | GaS | 0.281903 | 0.303207 |
| JVASP-19583 | AgNO2 | 0.282834 | 0.29056 |
| JVASP-27851 | Al2FeS4 | 0.28631 | 0.365806 |
| JVASP-60389 | Te3CrGe | 0.287274 | 0.287574 |
| JVASP-27940 | FePSe3 | 0.305458 | 0.295458 |
| JVASP-60253 | UTe3 | 0.310967 | 0.312571 |
| JVASP-60459 | UTe3 | 0.315493 | 0.365946 |
| JVASP-649 | MoSe2 | 0.315884 | 0.315184 |
| JVASP-648 | MoSe2 | 0.319225 | 0.318625 |
| JVASP-5950 | Sc2C | 0.323084 | 0.363274 |
| JVASP-13546 | VBr2 | 0.344168 | 0.344839 |
| JVASP-696 | CrS2 | 0.344733 | 0.376489 |
| JVASP-60566 | MoSe2 | 0.347102 | 0.423369 |
| JVASP-60237 | AuTe2 | 0.349614 | 0.349988 |
| JVASP-6130 | USe3 | 0.360518 | 0.339461 |
| JVASP-28143 | Tb2C | 0.367168 | 0.397985 |
| JVASP-768 | Ti2O | 0.371305 | 0.376805 |
| JVASP-19988 | AlN | 0.382007 | 0.350954 |
| JVASP-6226 | RuCl2O | 0.383991 | 0.378487 |
| JVASP-5980 | TaSe2 | 0.391839 | 0.414894 |
| JVASP-726 | CrS2 | 0.410568 | 0.443614 |
| JVASP-650 | MoSe2 | 0.419853 | 0.419055 |
| JVASP-5920 | TaSe2 | 0.425694 | 0.452691 |
| JVASP-27738 | I2Th | 0.427307 | 0.418828 |
| JVASP-8879 | VCl2 | 0.429254 | 0.430961 |
| JVASP-60603 | TaFeTe3 | 0.432357 | 0.485053 |
| JVASP-6862 | VS2 | 0.466561 | 0.493472 |
| JVASP-687 | GaSe | 0.477989 | 0.478089 |
| JVASP-6070 | TaS2 | 0.512431 | 0.543029 |
| JVASP-6667 | FeTe | 0.523567 | 0.520074 |
| JVASP-14431 | MnSe | 0.535549 | 0.528698 |
| JVASP-6880 | CrSb2 | 0.545075 | 0.541203 |
| JVASP-31361 | Cl2PdSe6 | 0.59646 | 0.697131 |
| JVASP-15708 | TlSb | 0.620986 | 0.824581 |
| JVASP-20415 | DyTe | 0.641184 | 0.299135 |
| JVASP-6754 | TiCl2 | 0.689358 | 0.700384 |
